# Engineered yeast provides rare but essential pollen sterols for honeybees

**DOI:** 10.1038/s41586-025-09431-y

**Published:** 2025-08-20

**Authors:** Elynor Moore, Raquel T. de Sousa, Stella Felsinger, Jonathan A. Arnesen, Jane D. Dyekjær, Dudley I. Farman, Rui F. S. Gonçalves, Philip C. Stevenson, Irina Borodina, Geraldine A. Wright

**Affiliations:** 1 Department of Biology, University of Oxford, Oxford, UK; 2 The Novo Nordisk Foundation Centre for Biosustainability, Technical University of Denmark, Kongens Lyngby, Denmark; 3 Natural Resources Institute, University of Greenwich, Chatham, UK; 4 Kew Science Trait Diversity and Function, Royal Botanic Gardens Kew, London, UK; 5 Present Address: Department of Biotechnology, Delft University of Technology, Delft, Netherlands

**Keywords:** Metabolic engineering, Agroecology, Entomology

## Abstract

Honeybees are important crop pollinators, but they increasingly face pollen starvation as a result of agricultural intensification and climate change^1^. Frequent flowering dearth periods and high-density rearing conditions weaken colonies, which often leads to their demise^2^. Beekeepers provide colonies with pollen substitutes, but these feeds do not sustain brood production because they lack essential sterols found in pollen^3,4^. Here we describe a technological advance in honeybee nutrition with wide-reaching impacts on global food security. We first measured the quantity and proportion of sterols present in honeybee tissues. Using this information, we genetically engineered a strain of the oleaginous yeast *Yarrowia lipolytica* to produce a mixture of essential sterols for bees and incorporated this yeast strain into an otherwise nutritionally complete diet. Colonies exclusively fed with this diet reared brood for significantly longer than those fed diets without suitable sterols. The use of this method to incorporate sterol supplements into pollen substitutes will enable honeybee colonies to produce brood in the absence of floral pollen. Optimized diets created using this yeast strain could also reduce competition between bee species for access to natural floral resources and stem the decline in wild bee populations.

## Abstract (web summary)

Genetic engineering of a yeast strain provides rare but essential pollen sterols for honeybee colonies.

## Main

Managed honeybees (*Apis mellifera*) are fundamental to modern agricultural systems and global food security because of the crucial pollination services they provide to crops. Honeybees consume floral pollen, which provides proteins, fats, carbohydrates and micronutrients^5^. Increasingly, a combination of changes in climate, land use and agricultural practices is limiting the access of honeybees to sufficient and diverse floral resources^1,6,7^. Nutritional deficiencies increase the susceptibility of bees to disease and colony collapse and contribute to the growing rate of honeybee colony losses^2,8^. Loss of pollinators in turn reduces crop yields and raises the costs of food production^9,10^. In the past 40 years, beekeepers have adopted the practice of feeding artificial pollen substitutes when natural forage is insufficient or when bees are kept at high densities. However, commercially available pollen substitutes composed of protein flour, sugars and oils are not nutritionally complete feeds for honeybees. Such diets are missing essential sterols found in floral pollen that are necessary for colony health and growth^4,11^.

Sterols are a structurally diverse class of tetracyclic triterpenoids that are ubiquitously important for eukaryotic cell function, including for membrane architecture, hormone biosynthesis and signalling^11–13^. Unlike other animals, insects and marine invertebrates have lost the ability to synthesize endogenous sterols and instead acquire them from the diet^12,13^. Most insects produce cholesterol (CHOL) by dealkylating dietary sterols^13,14^. By contrast, honeybees have lost the ability to dealkylate sterols and instead directly use sterols obtained from the diet as membrane inserts and as precursors for ecdysteroid hormones^15,16^. Most of the pollen sterols used by bees are not available in quantities that could be fed to bee colonies on a commercial scale, which makes it unfeasible to create a nutritionally complete feed that is a substitute for pollen.

## Honeybees obtain six sterols from pollen

**Fig. 1:**
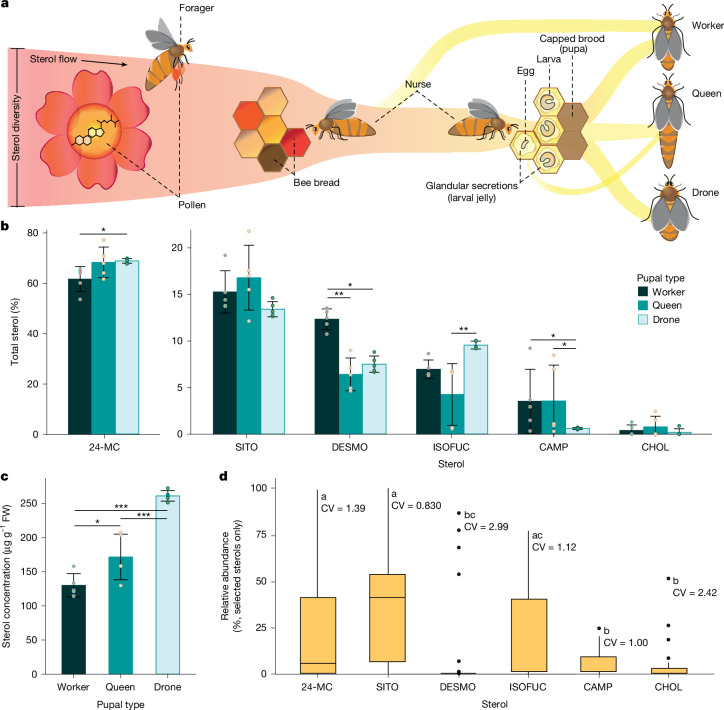
Sterol nutrition of a honeybee colony. **a**, Adult worker honeybees collect and eat floral pollen, which provides proteins, lipids and micronutrients. Pollen foraged from flowers is packed into cells of the wax comb with honey to form bee bread. Nurse bees consume bee bread and create glandular secretions (jelly), which include sterols required for development, to larvae. Larvae develop into pupae and eventually emerge as female workers, queens or drones (males). **b**, The relative abundance of each of the six key sterols found in pupae varies among pupal types (two-way quasi-binomial GLM, sterol × pupal type, *χ*^2^_10_ = 47.0, *P* < 0.0001, *n* = 5 biologically independent samples). Error bars represent the mean ± s.d. **c**, Total sterol concentration per fresh weight (FW) varies significantly among pupal types (one-way Gaussian GLM, *χ*^2^_2_ = 91.9, *P* < 0.0001, *n* = 5 biologically independent samples). Error bars represent the mean ± s.d. **d**, The relative abundance of selected sterols in pollen from bee-pollinated flowers (combined total (%), *n* = 41 biologically independent samples). Floral pollen varies significantly in the relative abundance of these sterols (one-way quasi-binomial GLM, *χ*^2^_5_ = 61.0, *P* < 0.001). The centre line of the box plots represents the median, the hinges represent the 25th and 75th percentiles, whiskers extend 1.5× the interquartile range (75th to the 25th percentile) from the hinges and data points beyond the whiskers are plotted individually. Coefficients of variation (CV) of each sterol are shown. Data were obtained from ref. ^18^ (Supplementary Data 1). Letters (**d**) and asterisks (**b**,**c**) denote significance (post hoc comparisons of estimated marginal means with Tukey adjustment, *P < 0.05, **P < 0.01, ***P < 0.001). Source data

**Extended Data Fig. 1:**
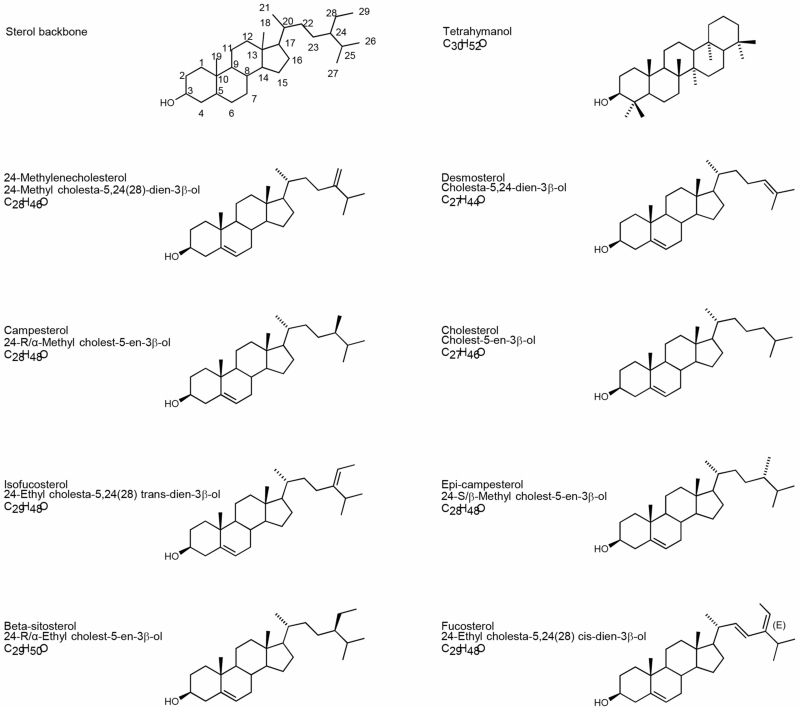
Chemical structures, names, and formulae of sterols relevant to this study. The basic sterol structure with numbering of the carbon atoms, as well as the structures, common names, systematic names, and chemical formulae of the main sterols referred to in this study.

Sterols are consumed by nurse-aged worker honeybees from stored pollen (bee bread). The sterols accumulate in their mandibular glands and are transferred to worker, queen and drone larvae through glandular secretions^17^ (Fig. 1a). Sterol diversity is high in pollen^18^, but only a few key sterols seem to be taken up by worker bees in the gut and transferred to the brood^19^ (Fig. 1a,b). To quantify the proportions of sterols found in honeybees, we analysed the sterol composition in pupal tissue from each pupal type. The Δ^5^-sterol 24-methylenecholesterol (24-MC; Extended Data Fig. 1) constituted 60–70% of sterols in the pupae (Fig. 1b). Five other minor sterols were consistently present: β-sitosterol (SITO), desmosterol (DESMO), isofucosterol (ISOFUC), campesterol (CAMP) and CHOL (Fig. 1b and Extended Data Fig. 1). The relative abundance of each sterol found in pupae varied among pupal types (two-way quasi-binomial generalized linear model (GLM), sterol × pupal type, *χ*^2^_10_ = 47.0, *P* < 0.0001; Fig. 1b). Drone pupae had 11.8% more 24-MC than worker pupae and 2.24-fold more ISOFUC than queen pupae. Worker pupae had at least 1.5-fold more DESMO than queen or drone pupae. CAMP was almost six times more abundant in worker and queen pupae than drone pupae.

**Extended Data Fig. 2:**
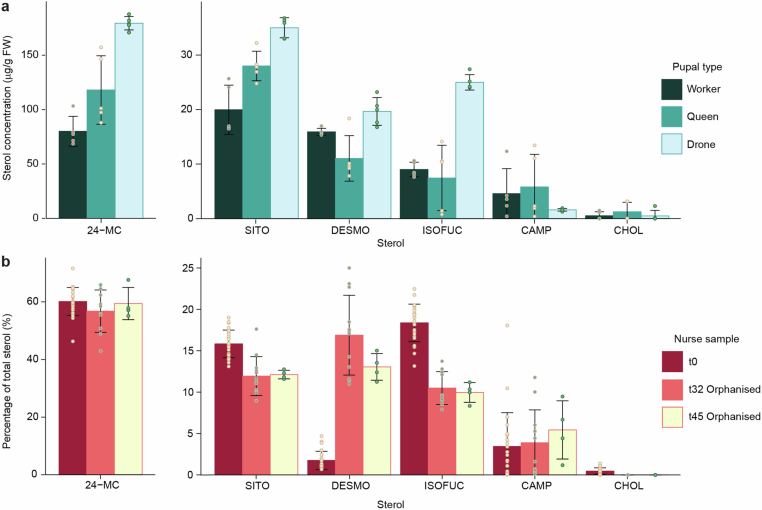
The major sterols in honeybee pupae from worker, queen, and drone bee pupal types and in nurse bees. **a**, Mean concentration of each of the major sterols per fresh weight of pupal tissue (*n* = 5 biologically independent samples). There was a significant interaction between sterol and pupal type (two-way Gaussian GLM, χ^2^(10) = 255, *P* < 0.0001). For each sterol, pairwise comparisons of estimated marginal means were carried out with Tukey *P*-value adjustment (*P < 0.05, **P < 0.01, ***P < 0.001). 24-MC was more concentrated in drone than queen and worker pupal tissue. Isofucosterol (ISOFUC) was also more concentrated in drone than queen and worker pupal tissue. Sitosterol (SITO) was more concentrated in drone than worker pupae. Error bars represent ± s.d. from the mean. **b**, Mean relative abundance of each of the major sterols in nurses from day 0, and orphanised top-up nurses from day 32 and day 45, given as a percentage of the total sterol (six pupae per sample, t0 nurses, *n* = 24 biologically independent samples); t32 orphanised nurses, *n* = 10; t45 orphanised nurses, *n* = 4) Error bars represent ± s.d. from the mean. Source data

The total sterol concentration in pupal tissues varied significantly among pupal types and ranged from 130 to 260 μg g^–1^ fresh tissue (Gaussian GLM, *χ*^2^_2_ = 91.9, *P* < 0.0001; Fig. 1c). The total sterol concentration in drone pupae was greater than in queen pupae and double that in worker pupae. The concentrations of individual sterols similarly varied among pupal types (Extended Data Fig. 2). Five of these sterols (SITO, CAMP, CHOL, ISOFUC and 24-MC) were among the six most common sterols found in pollen (SITO, CAMP, CHOL, ISOFUC, cycloartenol and 24-MC)^18^ (Supplementary Data 1). Analyses of previously published data^18^ showed that floral pollen varied significantly in the relative abundance of these sterols (quasi-binomial GLM, *χ*^2^_5_ = 61.0, *P* < 0.001). SITO had the highest median relative abundance (40.9%), followed by ISOFUC (9.50%) and 24-MC (5.84%), whereas CAMP, CHOL and DESMO were present at low levels (Fig. 1d). The coefficient of variation ranged from 0.830 (SITO) to 2.99 (DESMO; Fig. 1d).

## Engineered yeast produce pollen sterols

**Extended Data Fig. 3:**
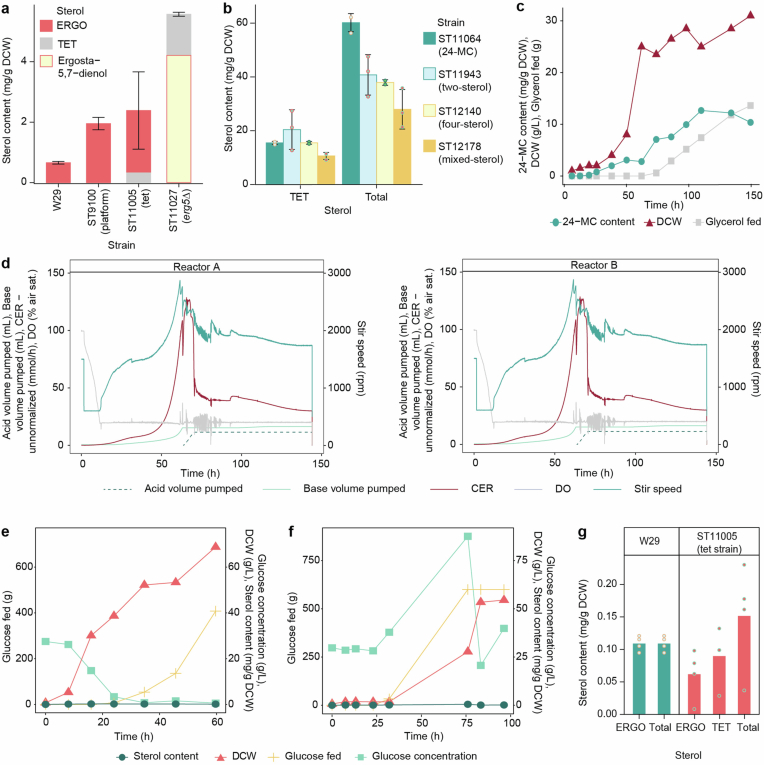
Characterisation and fermentation of engineered *Y. lipolytica* strains. **a**, Sterol content in W29, platform, TET and *erg5*Δ strains (*n* = 3 biologically independent samples). Error bars represent ± s.d. from the mean. **b**, TET, and total sterol content in *Y. lipolytica* strains engineered to produce a mixture of non-native sterols (*n* = 3 biologically independent samples). Error bars represent ± s.d. from the mean. **c**, Time course of sterol content, DCW and glycerol addition during fed-batch fermentation of the 24-MC strain in Ambr® 250 bioreactors (*n* = 2 biologically independent samples). **d**, Time course of operational parameters during fed-batch fermentation of the 24-MC strain in Ambr® 250 bioreactors (each plot represents one bioreactor). **e**, Time course of sterol content, DCW, glucose addition and glucose concentration in the broth during fed-batch fermentation of the W29 strain in 5-L bioreactors (data plotted is the average of two independent bioreactor cultivations). **f**, Time course of sterol content, DCW, glucose addition and glucose concentration in the broth during fed-batch fermentation of the TET strain in 5 L bioreactors (data plotted is the average of two independent bioreactor cultivations). **g**, Sterol composition of the W29 and TET strains after fed-batch fermentation in 5 L bioreactors (bioreactor cultivations were carried out in duplicate, sterol measurements from each bioreactor were carried out in technical duplicates). Source data

To produce the pollen sterols needed by bees, we used the oleaginous yeast *Y.* *lipolytica* as a host for sterol production. This yeast was chosen because of its high capacity for lipid accumulation and abundance of the sterol precursor acetyl-CoA^20–22^. Furthermore, we used a platform strain with improved mevalonate pathway flux towards farnesyl diphosphate to increase the supply of sterol precursors^23^. The platform strain is derived from the wild-type isolate W29 and it expresses a CRISPR-associated protein (Cas9) to enable genome editing. This strain also overexpresses four genes that encode mevalonate pathway enzymes: *HMG1*, *ERG12*, *IDI1* and *ERG20*. Genes involved in acetyl-CoA formation, namely ATP-citrate lyase 1 (*ACL1*), and acetyl-CoA synthase from *Salmonella enterica* (*ACS*) are also included. The platform strain produced almost three times more ergosterol (ERGO, 1.95 mg g^–1^ dry cell weight (DCW)) than the wild-type strain W29 (0.659 mg g^–1^ DCW; Extended Data Fig. 3a).

**Fig. 2:**
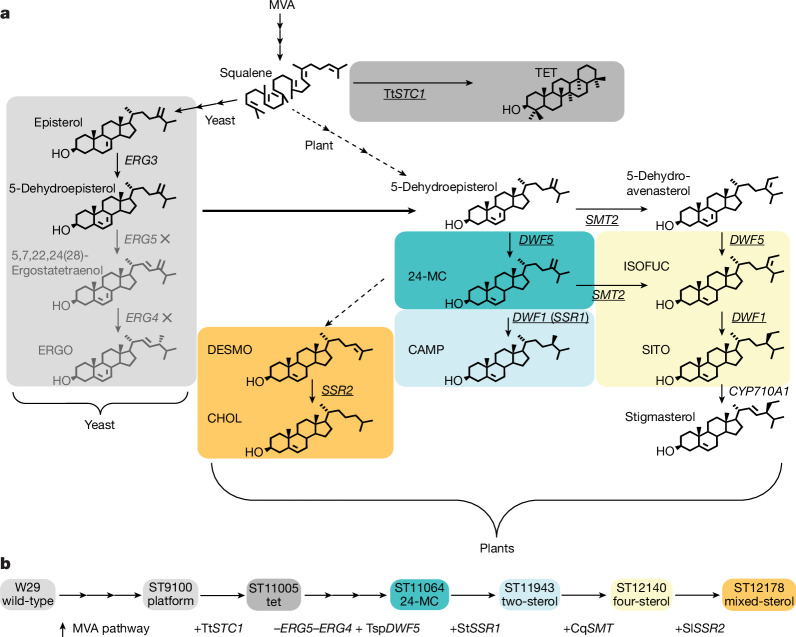
Biosynthesis pathways to produce the sterols used by bees in plants and engineered yeast. **a**, Schematic of the sterol biosynthesis pathway from mevalonate (MVA) in yeast and plants. The late steps in the pathways and the enzymes that catalyse these reactions are indicated: *ERG3* (Enzyme Commission (EC) number EC 1.14.21.6), C-5 desaturation; *ERG5* (EC 1.14.19.41), C-22 desaturation; *ERG4* (EC 1.3.1.71), Δ^24^ reduction; *SSR2* (EC 1.3.1.72), Δ^24(25)^ reduction; Tt*STC1* (EC 4.2.1.123), *T.* *thermophilia* squalene-tetrahymanol cyclase; *DWF5* (EC 1.3.1.21), Δ^7^ reduction; *DWF1* or *SSR1* (EC 1.3.1.72), Δ^24(28)^ reduction; *SMT2* (EC 2.1.1.143), C-28 methylation; *CYP710A1*, C-22 desaturation. A cross indicates a deleted gene, whereas an underline indicates a heterologous gene. **b**, Gene edits used to create *Y.* *lipolytica* strains capable of producing non-native sterols that are tailored for honeybees. Cq*SMT*, *SMT* from *C.* *quinoa*; Sl*SSR2*, *SSR2* from *S.* *lycopersicum*; St*SSR1*, *SSR1* from *S.* *tuberosum*; Tsp*DWF5*, *DWF5* from *Tetraselmis* sp.

The endogenous ERGO pathway of yeasts can be remodelled to synthesize alternative sterols (Fig. 2). However, previous attempts to produce non-native sterols in *Y.* *lipolytica* have struggled to achieve high specific yields or stereoselectivity (Supplementary Table 1). In *Saccharomyces cerevisiae*, genes involved in the late stages of the ERGO pathway are nonessential and alternative sterols can be taken up from the environment when ERGO biosynthesis is compromised, such as under anaerobic conditions^24,25^. However, most non-*Saccharomyces* yeasts, such as *Y.* *lipolytica*, lack sterol uptake transporters and do not exhibit flexible sterol auxotrophy^26^. For this reason, endogenous ERGO synthesis is essential, which makes deletion of latter ERGO pathway genes such as *ERG4* (which encodes Δ^24(28)^-sterol reductase) challenging in *Y.* *lipolytica*^27^.

To overcome this issue, we used a sterol surrogate. The previously characterized *STC1* gene from *Tetrahymena thermophilia* encodes a squalene-tetrahymanol cyclase that converts squalene to the pentacyclic triterpenoid tetrahymanol (TET; Extended Data Fig. 1), which can act as a substitute for ERGO function in yeasts^28–30^. TET is not anticipated to be detrimental to honeybees as pollen also contains diverse sterol intermediates and stanols that do not accumulate in honeybee tissues^18^. *T.* *thermophilia* *SCT1* was introduced into the platform yeast strain under the weak Pr*GPAT* promoter^31^ to drive minimal expression and to limit the diversion of carbon flux away from the sterol pathway. TET accounted for 14% of the sterols produced in the resulting TET strain (Extended Data Fig. 3a). By producing TET in *Y.* *lipolytica*, we were able to efficiently manipulate the sterol pathway. The subsequent elimination of ERGO production in our strains may have also helped to remove negative feedback inhibition on sterol biosynthesis.

**Fig. 3:**
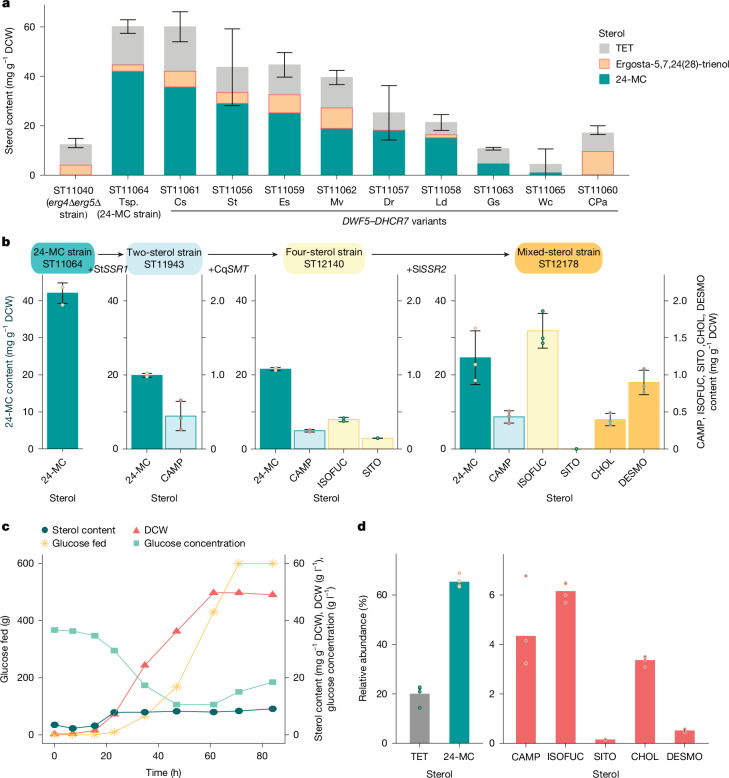
Engineering *Y.lipolytica* to produce key sterols required by honeybees. **a**, 24-MC production by engineered *Y.* *lipolytica* strains with different heterologous *DWF5* and *DHCR7* variants. Genes were selected from plants (*DWF5*), animals (*DHCR7*), algae, fungi and bacteria, including *Tetraselmis* sp. GSL018 (Tsp), *Coccomyxa subellipsoidea* (Cs) *S.* *tuberosum* (St), *Ectocarpus siliculosus* (Es), *Mortierella vertcillata* (Mv), *Danio rerio* (Dr), *Legionella drancourtii* (Ld), *G.* *soja* (Gs), *Waddlia chondrophila* (Wc) and ‘*Candidatus* Protochlamydia amoebophila’ (CPa). Strains were compared for the content of 24-MC, TET and the main pathway intermediate ergosta-5,7,24(28)-trienol (*n* = 3 biologically independent samples). Error bars represent the mean ± s.d. for the total sterol content. **b**, Sterol composition of *Y.* *lipolytica* strains progressively engineered to produce a mixture of additional sterols required by honeybees (*n* = 3 biologically independent samples). Error bars represent the mean ± s.d. TET and total sterol content are shown in Extended Data Fig. 3. **c**, Time course of sterol content, DCW, glucose addition and broth glucose concentration during fed-batch fermentation of the mixed-sterol strain in 5-litre bioreactors (bioreactor cultivations were carried out in duplicate, and measurements from each bioreactor were carried out in technical duplicates). **d**, Sterol composition of the mixed-sterol strain after 84 h of fermentation (bioreactor cultivations were carried out in duplicate, and sterol measurements from each bioreactor were carried out in technical duplicates). Time courses of 5-litre bioreactor fermentation of the TET and W29 strains and sterol content from 5-litre bioreactor fermentation of the mixed-sterol, TET and W29 strains are shown in Extended Data Fig. 3. Source data

We first engineered *Y.* *lipolytica* to produce the most important honeybee sterol, 24-MC. Redirection of the *Y.* *lipolytica* sterol pathway from ERGO to 24-MC required us to sequentially delete the endogenous genes *ERG4* and *ERG5* (which encode sterol C-22 desaturase) and introduce a heterologous *DWF5* or *DHCR7* gene (which encode Δ^7^-sterol reductase; Fig. 2). The *erg4*Δ*erg5*Δ strain was used to screen heterologous *DWF5* and *DHCR7* variants identified in the genomes of plants, animals, fungi and bacteria. Ten diverse variants were selected and expressed under the strong Pr*TEFintron* promoter^31^ (Fig. 3a). The strain that contained *DWF5* from *Tetraselmis* sp. (a green algal phytoplankton) produced the highest content of 24-MC (42.1 mg g^–1^ DCW) and accumulated less TET and ergosta-5,7,24(28)-trienol intermediates than the next highest producing strain, which expressed *DWF5* from *Coccomyxa subellipsoidea* (a unicellular green alga) (Fig. 3a).

The 24-MC-producing strain was subsequently engineered to create a mixed-sterol strain that produced small amounts of all the other sterols we observed in honeybees (Fig. 1b). We first introduced the previously characterized Δ^24(28)^-sterol reductase (encoded by *SSR1*) from potato *Solanum tuberosum*; this enzyme reduces the 24-MC side chain to produce CAMP^32^ (Fig. 2). By using a heterologous plant variant, we could regulate the expression level with the weak promoter Pr*DGA1* (ref. ^31^) and obtain the C-24α *R* epimer of CAMP found in plants rather than the C-24β *S* epimer as in ERGO (Extended Data Fig. 1). The resulting ‘two-sterol’ strain produced 24-MC (19.9 mg g^–1^ DCW) and a small amount of CAMP (0.445 mg g^–1^ DCW, Fig. 3b). We then constructed a strain that was further capable of producing C-24 ethyl sterols by introducing the C-28 sterol methyl transferase (encoded by *SMT*) from *Chenopodium quinoa* (quinoa) under the control of the Pr*GPAT* promoter. This ‘four-sterol’ strain produced ISOFUC (0.396 mg g^–1^ DCW), SITO (0.145 mg g^–1^ DCW), 24-MC (21.6 mg g^–1^ DCW) and CAMP (0.245 mg g^–1^ DCW, Fig. 3b).

To enable the production of CHOL and DESMO, the gene encoding Δ^24(25)^-sterol reductase (*SSR2*) from tomato *Solanum lycopersicum* was expressed under the Pr*GPAT* promoter in the four-sterol strain to generate the final mixed-sterol strain (Fig. 3b). Δ^24(25)^-sterol reductases are typically found in animals and reduce DESMO to CHOL but are also present in Solanaceous plants as part of the glycoalkaloid synthesis pathway^32^. When expressed in a 24-MC-producing *S.* *cerevisiae* strain, both DESMO and CHOL are produced^32^. This property was similarly observed in our final ‘mixed-sterol’ strain, which produced 24-MC (24.6 mg g^–1^ DCW), CAMP (0.433 mg g^–1^ DCW), ISOFUC (1.59 mg g^–1^ DCW), SITO (trace amounts), CHOL (0.396 mg g^–1^ DCW) and DESMO (0.898 mg g^–1^ DCW) during small-scale cultivation (Fig. 3b). The total detected sterol content decreased with each round of engineering from 60.2 mg g^–1^ DCW in the 24-MC strain to 28.0 mg g^–1^ DCW in the mixed-sterol strain (Extended Data Fig. 3b). The mixed-sterol strain was used for cultivation in bioreactors.

We investigated sterol and biomass production by cultivating the 24-MC strain in fed-batch mode in 250-ml controlled bioreactors. The 24-MC content reached 8.72–12.0 mg g^–1^ DCW, and the biomass reached 29.0–33.0 g DCW per litre after 150 h of fermentation (Extended Data Fig. 3c,d). To produce biomass for testing in honeybee diets, we cultivated the mixed-sterol strain in fed-batch mode in 5-litre bioreactors. After 84 h of fermentation, the sterol content was 8.46–9.76 mg g^–1^ DCW, and the cell density reached 48.3–49.8 g DCW per litre (Fig. 3c). The relative sterol content increased for the first 24 h and then remained stable. 24-MC constituted approximately 65% of the total sterol content (Fig. 3d). ISOFUC, CAMP, CHOL, DESMO and SITO together constituted 15% of the total sterol content, and TET contributed the remaining 20% (Fig. 3d). We also fermented the W29 strain and the TET strain in 5-litre bioreactors for use as controls in feeding experiments. The sterol content for these two strains reached 0.100–0.118 and 0.099–0.204 mg g^–1^ DCW, respectively (Extended Data Fig. 3e,f).

Previous studies of non-native sterol production in yeast have achieved up to 36 mg g^–1^ DCW epi-campesterol in *Y.* *lipolytica* and 19.3 mg g^–1^ DCW epi-ergosterol in *S.* *cerevisiae* under fermentation conditions (Supplementary Table 1). We initially achieved 42.1 mg g^–1^ DCW of 24-MC during small-scale cultivation, which is eightfold more sterol per unit biomass than vegetable oils or floral pollen^18,33^. This result demonstrates the utility of the pre-engineered platform strain and the potential of *Y.* *lipolytica* as a host for sterol production. However, the sterol content reduced with each engineering step of the strain and during cultivation scale-up. Reductions in sterol content have been observed in other studies of similar strains during bioreactor cultivation^34,35^. Nevertheless, pathway optimization, capacity for sterol storage and growth conditions for lipid accumulation can be further improved to increase the sterol content in *Y.* *lipolytica*^36^. Successful strategies in yeast include regulation of cellular sterol homeostasis and enzyme relocalization to lipid droplets^37,38^.

## Pollen sterols support brood production

The resulting yeast biomass from fermentation was dried and ground to create diets that could be tested in semi-field feeding trials with honeybee colonies. Dried yeast biomass was incorporated at 20% w/w into an artificial base-diet formulation that contained additional proteins, fats, vitamins and minerals required by honeybees. The following diets were tested: a mixed-sterol strain diet (MxSt), a TET strain diet (Tet), a W29 strain diet (WT) and a no yeast base diet (base). The latter three were used as control diets. Because the mixed-sterol strain produced only trace amounts of SITO, all yeast-based diets were supplemented with a commercial phytosterol mix, which contained approximately 12.0% w/w SITO (Supplementary Table 7). The base diet was also supplemented with a commercial phytosterol mix to match the total sterol concentration in the MxSt diet (0.340% w/w).

**Fig. 4:**
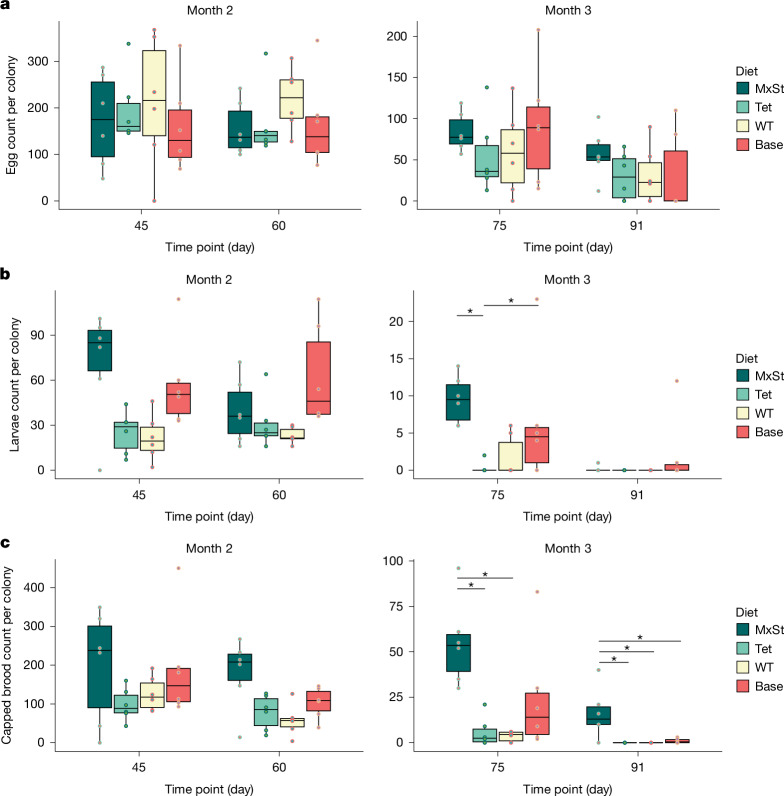
Semi-field trials to examine the impact of dietary sterols on honeybee colonies. **a**–**c**, Number of eggs (**a**), larvae (**b**) and capped brood cells (**c**) counted per colony for each treatment group for the last 2 months of the feeding trials. Eggs, larvae and capped brood cells were counted in each colony at 15-day intervals (*n* = 6 biologically independent colonies). Colonies were provided with a MxSt diet or one of three control diets: a Tet diet, a WT diet or a base diet. For all panels, the centre line of the box plots represent the median, the hinges represent the 25th and 75th percentiles, whiskers extend 1.5× the interquartile range (25th to the 75th percentile) from the hinges and data points beyond the whiskers are plotted individually. Statistical analysis was performed by fitting two-way negative binomial GLMMs followed by post hoc comparisons of estimated marginal means at each time point with Tukey adjustment (*P < 0.05, **P < 0.01, ***P < 0.001). The number of eggs varied with time but not diet treatment group (diet + time, time: *χ*^2^_3_ = 48.3, *P* < 0.0001; diet: *χ*^2^_3_ = 0.782, *P* = 0.854). The number of larvae varied with diet treatment group (diet × time, *χ*^2^_9_ = 18.4, *P* = 0.0304). The number of capped brood also varied with diet treatment group (diet × time, *χ*^2^_9_ = 30.0, *P* = 0.0004). Brood counts from the first month of the feeding trials are shown in Extended Data Fig. 4. Note that the scales for months 2 and 3 differ, as brood production waned at the end of summer. Source data

**Extended Data Fig. 4:**
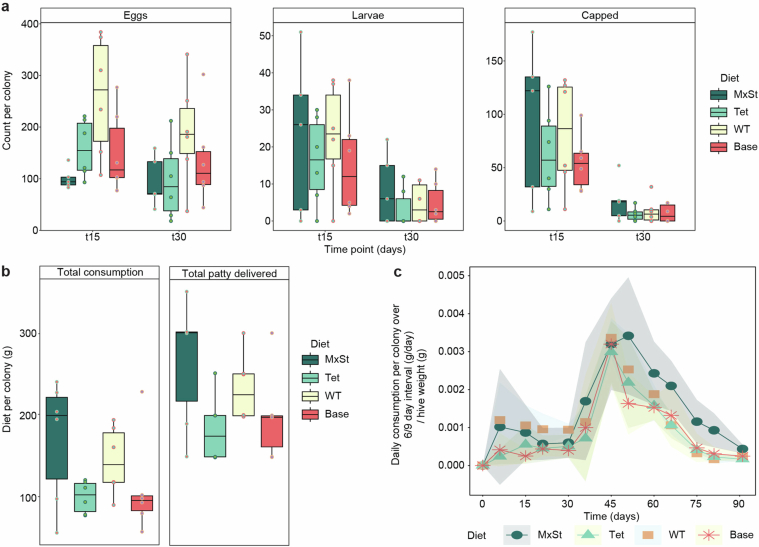
Initial colony brood status and diet consumption. **a**, Number of eggs, larvae and capped brood cells counted per hive for each treatment group for the first month of the feeding trials. Eggs, larvae, and capped brood cells were counted in each colony at 15-day intervals (*n* = 6 biologically independent colonies). The centre line of the boxplots represent the median, the hinges represent the 25^th^ and 75^th^ percentiles, whiskers extend 1.5 x the interquartile range (75^th^ – 25^th^ percentile) from the hinges, whilst data points beyond the whiskers are plotted individually. **b**, The total consumption (one-way Gaussian GLM, χ^2^(3) = 3.15, *P* = 0.370) and total diet provided (one-way Gaussian GLM, χ^2^(3) = 5.74, *P* = 0.1252) to each colony over the course of the feeding trials did not vary significantly between treatment groups (*n* = 6 biologically independent colonies). The centre line of the boxplots represent the median, the hinges represent the 25^th^ and 75^th^ percentiles, whiskers extend 1.5 x the interquartile range (75^th^ – 25^th^ percentile) from the hinges, whilst data points beyond the whiskers are plotted individually. **c**, Time course of diet consumption rate normalised by hive weight (*n* = 6 biologically independent colonies). The normalised consumption rate varied with time (two-way Gaussian GLMM, χ^2^(12) = 526, *P* < 0.0001) and diet treatment group (χ^2^(3) = 15.8, *P* = 0.0012). *Post hoc* pairwise comparison of estimated marginal means revealed that consumption rate was higher for colonies provided with the MxSt diet than those provided with the Tet (*P* = 0.0087) or Base (*P* < 0.0001) diets at t51. At t66, consumption rate for colonies provided with the MxSt diet was significantly greater than those provided with the Tet (*P* = 0.0388) or WT (*P* = 0.0385) diets. Error ribbons represent ± s.d. from the mean. Source data

**Extended Data Fig. 5:**
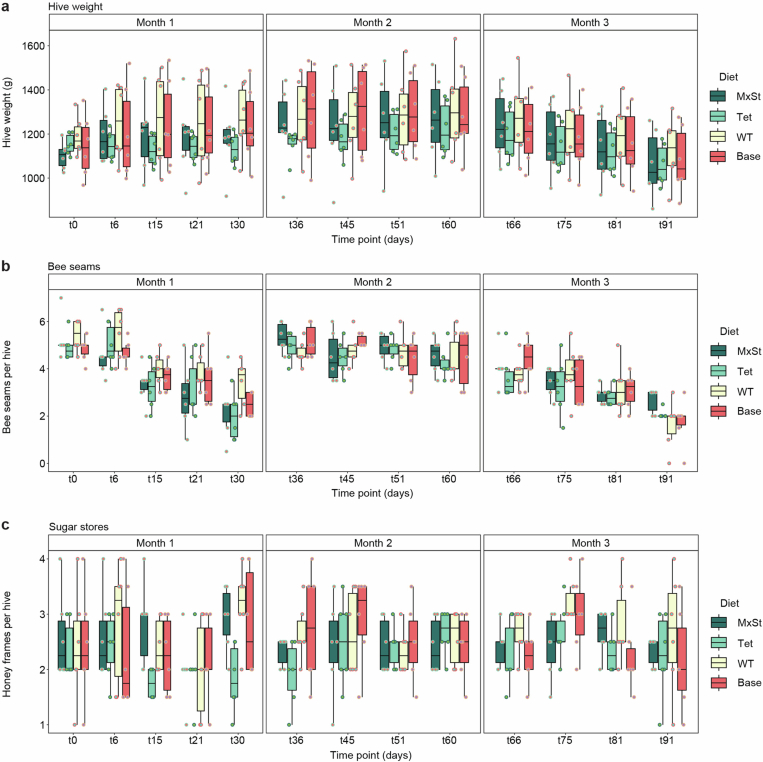
Feeding trial hive monitoring. **a**, Variation in hive weight during feeding trials (*n* = 6 biologically independent colonies). Hive weight varied with time but not diet treatment group (two-way Gaussian GLMM, time: χ^2^(12) = 176, *P* < 0.0001, diet: χ^2^(3) = 1.76, *P* = 0.623). **b**, Variation in number of bee seams during feeding trials (*n* = 6 biologically independent colonies). Bee seams varied with time but not diet treatment group (two-way Poisson GLMM (fitted to doubled count values), time: χ^2^(12) = 144, *P* < 0.0001; diet: χ^2^(3) = 1.38, *P* = 0.710). **c**, Variation in sugar stores during feeding trials (*n* = 6 biologically independent colonies). Sugar stores did not vary significantly with time or diet treatment group (two-way Poisson GLMM (fitted to doubled count values), time: χ^2^(12) = 8.67, *P* = 0.731; diet: χ^2^(3) = 3.11, *P* = 0.375). For all panels, the centre line of the boxplots represent the median, the hinges represent the 25^th^ and 75^th^ percentiles, whiskers extend 1.5x the interquartile range (75^th^ – 25^th^ percentile) from the hinges, whilst data points beyond the whiskers are plotted individually. Source data

**Extended Data Fig. 6:**
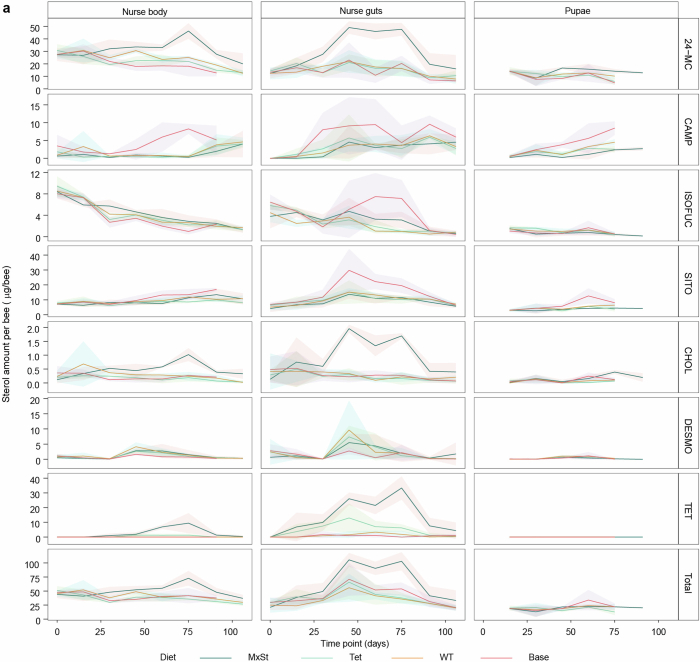
Time course of sterol content in nurse carcasses, nurse guts and pupae. **a**, Lines represent the average sterol content per bee (*n* = 3 biologically independent samples) for each treatment group (‘mixed-sterol strain diet’ (MxSt), ‘tetrahymanol strain diet’ (Tet), ‘W29 strain diet’ (WT), or ‘no yeast base diet’ (Base)). Error ribbons represent ± s.d. from the mean. Full statistical analysis results are presented in Supplementary Data 2. From t45 to t75, there was significantly more sterol in the guts of nurses from the MxSt treatment group than those from any other treatment group (*post hoc* pairwise comparison of estimated marginal means, all *P* values < 0.0133). By t75 there was more sterol in the nurse bodies from the MxSt treatment group than all other treatment groups (all *P* values < 0.0001). Total sterol content in the pupae varied significantly with time but not diet treatment group (two-way Gaussian GLMM, time: χ^2^(4) = 13.3, *P* = 0.0098; diet: χ^2^(3) = 3.50, *P* = 0.321). There was more TET in the nurse guts from the MxSt diet treatment group than all other treatment groups from t45-t60 (all *P* values < 0.0005) and more TET in the nurse bodies from the MxSt diet treatment group than all other treatment groups at from t60-t75 (all *P* values < 0.0001). Only trace amounts of TET were detected in the pupae. Source data

**Extended Data Fig. 7:**
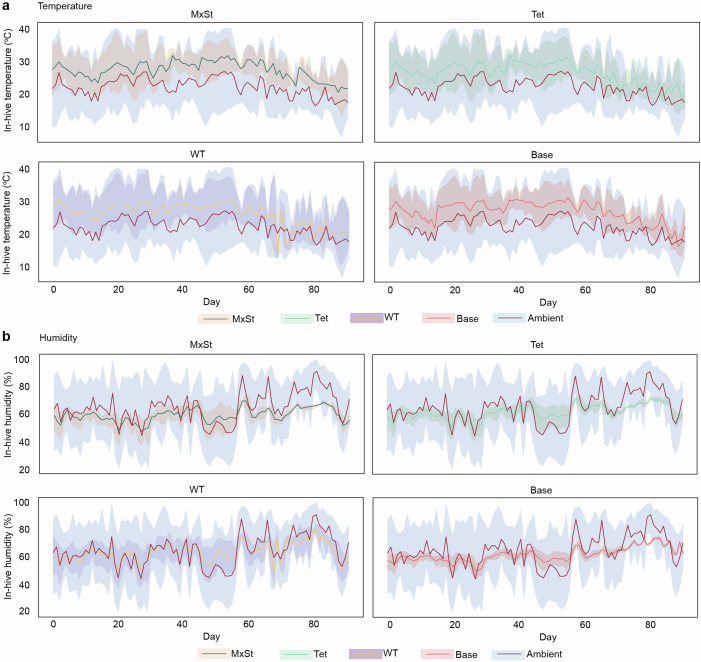
Temperature and humidity monitoring during feeding trials. Time course of temperature (**a**) and humidity (**b**) in mini-nucleus hives from each treatment group (‘mixed-sterol strain diet’ (MxSt; *n* = 5 independent sensors in different hives), ‘tetrahymanol strain diet’ (Tet; *n* = 6 independent sensors in different hives), ‘W29 strain diet’ (WT; *n* = 5 independent sensors in different hives), or ‘no yeast base diet’ (Base; *n* = 5 independent sensors in different hives)), and in the glasshouse (ambient; *n* = 5 independent sensors). Lines represent the daily mean recordings. Ribbons depict the average maximum and minimum daily values. Note: extreme high temperatures occurred during days 26–29 and during days 49–55 which impacted colony brood production.

We used a semi-field method with small colonies of honeybees to measure the impact of the different diets on brood production in the absence of incoming pollen. We conducted feeding trials over 3 months and monitored diet consumption, sterol composition, colony size and brood status (Fig. 4 and Extended Data Figs. 4–6). Every 15 days, we counted broods at the egg, larval and pupal (capped cell) stages in each colony to assess the ability of each treatment to support brood production and development. In the first 45 days, an extreme heatwave resulted in a reduction in bees of all ages across all colonies (Extended Data Fig. 7). Hence, we supplemented colonies with extra nurse bees on days 32 and 45. Nurse bees have endogenous sterols that can be used to feed larvae^3,16,19^ (Fig. 2). For this reason, we analysed the brood data starting from day 45, when we stopped adding nurse bees.

**Extended Data Fig. 8:**
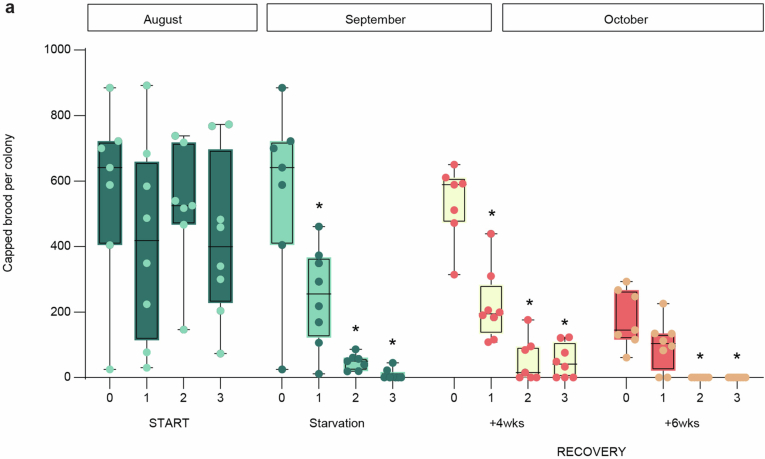
Pollen starvation combined with seasonal changes in a semi-field experiment leads to failed brood production. **a**, Brood production (count of capped cells/colony) was equal across all four treatments (0, 1, 2, 3, x-axis) at the start of the experiment (one-way ANOVA, F_3, 26_ = 0.594, *P* = 0.625). Colonies starved for 1, 2 or 3 weeks (x-axis) succumbed to seasonal reduction in brood production at a rate faster than the control (two-way ANOVA, time*treatment, F_6,77_ = 5.06, *P* < 0.001). The control (not starved, 0), like the other groups, exhibited a decline in brood production at the end of the season in October (Sidak, START vs +6wks for the control group, *P* = 0.003). The x-axis indicates the treatment (i.e. the number of weeks that the colonies were starved of pollen, 0, 1, 2, 3) over the three periods of the experiment (Start, Starvation period where no pollen was provided, and Recovery period of 4 weeks of pollen feeding or 6 weeks of pollen feeding. These data indicate that even when starved colonies are provided with pollen, it is not sufficient for them to recover from previous nutrient deprivation. * indicates a significant post-hoc comparison within time point of treatment vs control (*P* < 0.01). *n* = 8 biologically independent colonies for the 1 and 3 week starvation treatments, *n* = 7 biologically independent colonies for the pollen and 2 week starvation treatments. The centre line of the boxplots represent the median, the hinges represent the 25^th^ and 75^th^ percentiles, whiskers extend to the minimum and maximum data points.

The number of eggs varied with time but not diet treatment group (two-way negative binomial generalized linear mixed model (GLMM), diet + time, time: *χ*^2^_3_ = 48.3, *P* < 0.0001; diet: *χ*^2^_3_ = 0.782, *P* = 0.854; Fig. 4a). Feeding trials continued until mid-September when egg production in colonies begins to decline going into autumn. As such, there was a threefold reduction in egg laying from day 45 to day 91 and a corresponding decrease in the numbers of larvae, capped brood and adults (Fig. 4 and Extended Data Fig. 5). Overall, the number of larvae varied among the diet treatment groups, and the effect of the diet became apparent by day 75 (two-way negative binomial GLMM, diet × time, *χ*^2^_9_ = 18.4, *P* = 0.0304; Fig. 4b). At day 75, there were significantly more larvae in the MxSt diet treatment group than the Tet diet treatment group (post hoc comparison of estimated marginal means with Tukey adjustment, *P* = 0.0109). The base diet treatment group also had more larvae than the Tet diet treatment group (*P* = 0.0295). By day 91, larval counts were low in all colonies. The low brood production at this time point in the MxSt treatment was due to a seasonal effect that is also observed when colonies are fed with pollen (Extended Data Fig. 8).

The type of diet significantly affected the brood reared to pupation. As time passed, the impact of the diet on bees increased, such that colonies fed with the MxSt diet were more likely to rear larvae to the viable pupal stage (two-way negative binomial GLMM, diet × time, *χ*^2^_9_ = 30.0, *P* = 0.0004; Fig. 4c). By day 75, there were significantly more pupae in the MxSt diet treatment group than the Tet (*P* = 0.0333) and WT (*P* = 0.0230) diet treatment groups. By the end of the experiment (day 91), there were also significantly more pupae in the MxSt diet treatment group than the base (*P* = 0.0328), Tet (*P* = 0.020) and WT (*P* = 0.020) diet treatment groups. There were still 10–40 capped brood cells in all but 1 colony in the MxSt diet treatment group. By contrast, there were 1–3 capped brood cells in 3 colonies in the base diet treatment group and none in the control yeast diet groups. The data for the control diets (which do not have the required sterols) were similar to our observations of colonies starved of pollen, whereby the rate of brood production is a function of the duration of nutrient starvation (Extended Data Fig. 8). Eventually, in all colonies fed with diets that lacked the required sterols (base, Tet and WT diets), the nurse bees did not have sufficient levels of key corporeal sterols necessary for rearing brood.

**Extended Data Fig. 9:**
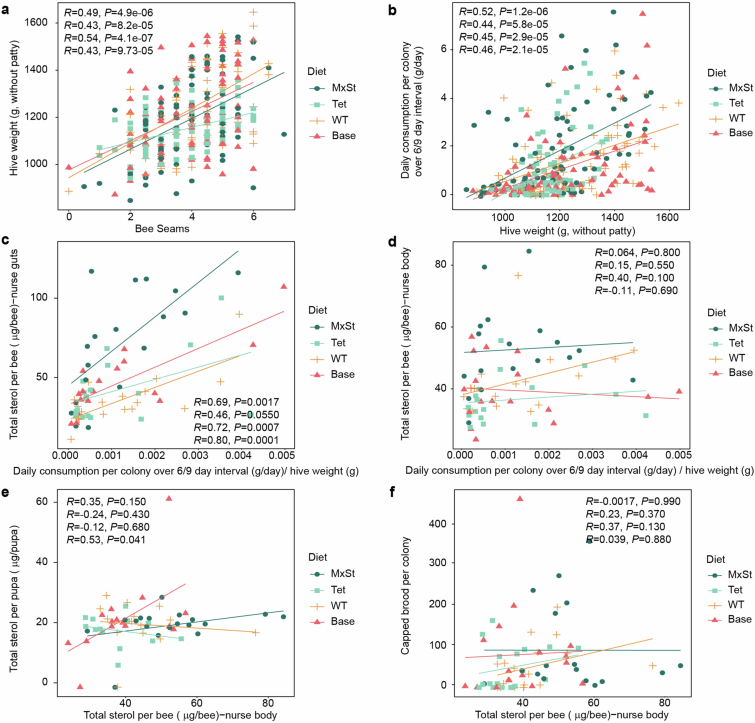
Regression analysis of feeding trial metrics. **a**, Hive weight varied significantly with bee seams (two-way Gaussian GLM, χ^2^(1) = 83.9, *P* < 0.0001) and diet treatment group (χ^2^(3) = 12.9, *P* = 0.0049, ‘mixed-sterol strain diet’ (MxSt), ‘tetrahymanol strain diet’ (Tet), ‘W29 strain diet’ (WT), or ‘no yeast base diet’ (Base); *n* = 6 biologically independent colonies). **b**, Consumption rate varied significantly with hive weight (two-way Gaussian GLM, χ^2^(1) = 85.5, *P* < 0.0001) and diet treatment group (χ^2^(3) = 15.1, *P* = 0.0018; *n* = 6 biologically independent colonies). **c**, Total sterol per nurse gut varied significantly with normalised consumption rate (two-way Gaussian GLM, χ^2^(1) = 43.6, *P* < 0.0001) and diet treatment group (χ^2^(3) = 36.2, *P* < 0.0001; *n* = 6 biologically independent colonies). **d**, Total sterol per nurse body did not vary significantly with normalised consumption rate (two-way Gaussian GLM, χ^2^(1) = 0.844, *P* = 0.358) but did vary with diet treatment group (χ^2^(3) = 23.1, *P* < 0.0001; *n* = 6 biologically independent colonies). **e**, Sterol per pupa vs sterol per nurse body. There was a significant interaction between total sterol per nurse body and diet treatment group (two-way Gaussian GLM, χ^2^(3) = 9.06, *P* = 0.0285; *n* = 6 biologically independent colonies). **f**, Capped brood cell counts did not vary with total sterol per nurse (two-way negative binomial GLM, χ^2^(1) = 1.08, *P* = 0.300) or diet treatment group (χ^2^(3) = 1.83, *P* = 0.609; *n* = 6 biologically independent colonies). The Pearson correlation coefficient (*R*) and *P*-values are shown. Source data

Although the number of pupae correlated with the diet given, sterol levels in the surviving pupae varied less than in the nurse bees and were not significantly different among the treatment groups (Gaussian GLMM, diet × time, time: *χ*^2^_4_ = 14.9, *P* = 0.005; diet: *χ*^2^_3_ = 4.39, *P* = 0.222; Extended Data Fig. 6). Moreover, sterol levels in the bodies of nurse bees showed less variation than the gut contents (Extended Data Fig. 6). However, neither the sterol content of the pupae nor the number of pupae correlated with the total sterol content in the bodies of nurse bees (Extended Data Fig. 9). In the MxSt and Tet diet treatment groups, TET accumulated in the guts of nurse bees (up to 42.4 μg per bee) but to a lesser extent in the bodies (up to 18.7 μg per bee) and not at all in the pupae (Extended Data Fig. 6).

Pollen contains diverse sterols, including cyclopropyl intermediates and stanols^18^, but our data indicated that only the Δ^5^-terminal intermediates of the sterol pathway tended to accumulate in honeybees. In yeast-feeding experiments with our mixed-sterol strain, nurse workers transferred only the key sterols produced by the yeast (24-MC, ISOFUC, DESMO, CAMP, SITO and CHOL) to the brood. Two diets contained the sterol surrogate TET, but this was not found in the brood. This finding indicates that bees have selective mechanisms for enriching larval jelly with the key sterols. Our data validate previous findings^11^ that honeybees probably require multiple sterols for continued brood production. The most important sterols may be 24-MC and CHOL, as colonies fed sterol-free diets supplemented with 24-MC or CHOL produce more pupae than when supplemented with SITO or CAMP^11^.

We observed a clear benefit to colonies that were fed the MxSt diet, which contains less than 0.5% w/w sterol. Understanding the full impact on the health and performance of honeybees fed with our MxSt yeast would require a long-term field study with standard size colonies. Yeast biomass is an additional valuable source of proteins, lipids and vitamins^22,39^. Hence, it may be preferable to favour biomass over sterol yield when optimizing fermentation parameters to produce biomass for feed. We speculate that further engineering of lipid, protein and terpenoid profiles to produce nutrients such as beneficial fatty acids, antioxidants, carotenoids and fragrances^40–42^ could extend the suitability of *Y.* *lipolytica* biomass as a honeybee feed. *Y.* *lipolytica* biomass is regarded as safe for inclusion in food and feed, and engineered biomass has been established as feed in aquaculture^39,43,44^. The direct use of inactivated yeast biomass in the diet circumvents the need for sterol extraction, which is expensive and inefficient, and minimizes downstream processing. Such technology could also provide an avenue for the development of feeds for other farmed arthropods.

## Methods

### Strains, culture conditions and chemicals

*Escherichia coli* strain DH5α was used for plasmid construction. *E.* *coli* was grown at 37 °C and 300 r.p.m. in lysogeny broth (LB) liquid medium and at 37 °C on plates of LB solid medium supplemented with 20 g l^–1^ agar. Ampicillin was supplemented at a concentration of 100 mg l^–1^ for plasmid selection.

The *Y.* *lipolytica* W29 strain (MATa, Y-63746 ARS Culture Collection, The National Center for Agricultural Utilization Research) and the W29-derived platform strain ST9100 (MATa ku70∆::PrTEF1-cas9-TTef12::PrGPD-dsdA-TLip2 IntC_2-HMG<-PrGPD-PrTefInt->ERG12 pCfB8823 IntC_3-SeACS<-PrGPD-PrTefInt->YlACL1 IntD_1-IDI1<-PrGPD-PrTefInt->ERG20, a mevalonate-upregulated strain) have been previously described^23^. The platform strain ST9100 was used to construct the sterol-producing strains. Details for all of the strains used in this study are provided in Supplementary Table 2.

*Y.* *lipolytica* was grown at 30 °C on yeast extract peptone dextrose (YPD) medium containing 10 g l^–1^ yeast extract, 20 g l^–1^ peptone and 20 g l^–1^ glucose, supplemented with 20 g l^–1^ agar for preparation of solid medium. For selection, either nourseothricin (250 mg l^–1^) or hygromycin (400 mg l^–1^) was added to the medium. Cultivation of strains for sterol production was performed in YPD medium containing 80 g l^–1^ glucose. Chemicals were obtained, unless indicated otherwise, from Sigma-Aldrich or Merck. Nourseothricin was purchased from Jena BioScience.

### Plasmid construction

The following coding sequences for enzymes were codon-optimized for *Y.* *lipolytica* and synthesized as GeneArt Strings DNA fragments by Thermo Fisher Scientific: Δ^7^-sterol reductase from *S.* *tuberosum* (St*DWF5*, GenBank accession: BAQ55276.1), *D.* *rerio* (Dr*DHCR7*, accession: NP_958487.2), *L.* *drancourtii* (Ld*DWF5*, accession: FJ197317.1), *E.* *siliculosus* (Es*DWF5*, accession: CBN77313.1), ‘*Candidatus* Protochlamydia amoebophila’ (CPa*DWF5*, accession: KIC71363.1), *C.* *subellipsoidea* (Cs*DWF5*, accession: XM_005650286.1), *M.* *vertcillata* (Mv*DWF5*, accession: KFH65691.1), *G.* *soja* (Gs*DWF5*, accession: XP_028244742.1); *Tetraselmis* sp. GSL018 (Tsp*DWF5*, accession: JAC78771.1) and *W.* *chondrophila* (Wc*DHCR7*, accession: ADI39181.1); squalene-tetrahymanol cyclase from *T.* *thermophilia* (Tt*STC1*, accession: XP_001026696.2); Δ^24(25)^-sterol reductase from *S.* *lycopersicum* (Sl*SSR2*, accession: BAQ55273.1); C-28 sterol methyl transferase from *C.* *quinoa* (Cq*SMT*, accession: XP_021737090.1); and Δ^24(28)^-sterol reductase from *S.* *tuberosum* (St*SSR1*, accession: AB839749.1). The codon-optimized sequences are listed in Supplementary Table 3.

The plasmids, BioBricks and primers used in this study are listed in Supplementary Tables 4–6. BioBricks were amplified by PCR using Phusion U polymerase (Thermo Scientific). BioBricks were assembled into EasyCloneYALI vectors with uracil-specific excision reagent (USER) cloning^31^. For marker-mediated gene deletion, upstream and downstream homology arms for relevant genes were synthesized as BioBricks by PCR amplification from the genomic DNA of the platform strain ST9100. Knockout constructs were assembled from BioBricks through USER reactions as detailed in Supplementary Table 5. USER reactions were transformed into *E.* *coli*, and correct assemblies were verified by Sanger sequencing (Eurofins).

### Yeast transformation

The yeast vectors were integrated into different previously characterized intergenic loci in the *Y.* *lipolytica* genome as previously described^31^. Integration vectors were digested with NotI enzyme (New England BioLabs) before lithium acetate transformation, as previously described^31^. Correct integration was verified by colony PCR using Taq DNA polymerase master mix RED (Ampliqon) with vector-specific primers and primers complementary to the genomic region adjacent to the integration site^31^.

For marker-mediated gene deletion, transformants were selected on YPD plates supplemented with antibiotic, and correct transformants were confirmed by colony PCR. Marker removal was performed by transformation of the strains with a Cre-recombinase episomal vector^31^. Marker removal was confirmed by colony PCR.

### Yeast cultivation

Yeast strains were inoculated into 2.5 ml YPD in 24-deep-well plates with air-penetrable lids (EnzyScreen). The plates were incubated at 30 °C with 300 r.p.m. agitation for 24 h. The optical density at 600 nm (OD_600_) was measured with an Implen P300 NanoPhotometer. The cultures were then diluted to an OD_600_ of 0.1 in 2.5 ml fresh YPD medium with 80 g l^–1^ glucose and grown for a further 72 h at 30 °C with 300 r.p.m. agitation. All cultivations were performed in triplicate. DCW was measured at the end of cultivation, whereby 1 ml of culture broth was transferred to a preweighed 2 ml microcentrifuge tube, centrifuged (3,000*g*, 5 min) and the supernatant was discarded. The cells were then washed twice with deionized water (1 ml). The cell pellet was dried at 60 °C for 7 days before the final weight was measured.

### Sterol analysis

For sterol extraction from yeast, 1 ml of culture broth was transferred to a 2 ml microcentrifuge tube, centrifuged and the supernatant was discarded. The cells were washed twice with deionized water (1 ml). The cell pellet was resuspended in 10% w/v methanolic potassium hydroxide (500 μl) and transferred to a 1 ml glass vial for saponification. The suspension was incubated at 70 °C for 2 h with vortexing at 15-min intervals. The saponified samples were then vortexed and spiked with 50 μl of internal standard (1 mg ml^–1^ epicoprostanol in absolute ethanol). Next, 500 μl *n*-hexane was added to each sample for extraction of the free sterol component. Samples were vortexed and the organic phase transferred to a 2 ml microcentrifuge tube. The extraction step was repeated in a further 500 μl *n*-hexane. The combined hexane phases were left overnight at room temperature for evaporation of the solvent. Sterol crystals remained in the tube.

For sterol analysis of the diets, each diet was sampled three times into preweighed 20 ml glass vials, and the weight of each sample was recorded. For sterol extraction from honeybee tissues, samples were first dried by freeze drying. Samples were dried at −48 °C under vacuum for 4 days. Dried samples were weighed and stored at −80 °C. For saponification, samples were first broken up with a spatula. For gut samples (in 2 ml microcentrifuge tubes), samples were suspended in 500 μl 10% w/v methanolic potassium hydroxide. For honeybee tissue samples (in 20 ml vials; pupae, nurse carcasses and queens), samples were suspended in 2.5 ml 10% w/v methanolic potassium hydroxide. Diet samples (in 20 ml vials) were suspended in 5 ml 10% w/v methanolic potassium hydroxide. Samples were incubated at 70 °C for 2 h in a water bath, with vortexing at 30–60 min intervals. The saponified samples were then spiked with 50 μl (gut samples) or 100 μl (diet, pupae, nurse carcasses and queen samples) of internal standard (1 mg ml^–1^ epicoprostanol in absolute ethanol). For extraction of the free sterol component, 500 μl (gut samples), 2.5 ml (pupae, nurse bee carcasses and queen samples) or 5 ml (diet samples) *n*-hexane was added to each sample. Samples were vortexed and the organic phase transferred to a 2 ml (gut samples) or 7 ml (diet, pupae, nurse bee carcasses and queen samples) glass vial. The extraction step was repeated and the combined hexane phases were left overnight at room temperature for evaporation of the solvent. The resulting extracts were resuspended in 500 μl (gut samples) or 1 ml (diet, pupae, nurse bee carcasses and queen samples) *n*-hexane and vortexed. From each sample, a subsample of 250 μl was transferred to a 1.5 ml microcentrifuge tube and left at room temperature overnight for final drying.

Sterols were resuspended in 500 μl pyridine that contained 20 μl *N,O*-bis(trimethylsilyl)acetamide (Merck) and incubated for 4 h at 50 °C and then briefly vortexed before direct injection into an Agilent Technologies 8860 gas chromatograph connected to an Agilent Technologies 5977 MSD mass spectrometer (for gas chromatography–mass spectrometry). Samples were eluted over an Agilent HP-5MS column using a splitless injection at 250 °C with a standard gas chromatography program at 170 °C for 1 min, ramped to 280 °C at 20 °C min^−1^ and monitoring between 50 and 550 AMU.

Sterols were identified by comparing their retention time relative to CHOL and mass spectra data available from the National Institute of Standards and Technology mass spectral library per a previous study^18^. Sterol identity in the final strain ST12178 was confirmed through comparison with authentic standards. Sterols were quantified by calculating the ratio of the peak area of the targeted sterol to that of the internal standard. The mass of each sterol in the sample was obtained by multiplying the ratio with the mass of the internal standard. Compound identification (using target ions) and quantification were carried out using ChemStation Enhanced Data Analysis (v.E.01.00).

### Bioreactor fed-batch cultivation

The Ambr 250 system (Sartorius Stedim Biotech) was used to carry out 250 ml fed-batch fermentation in duplicate. The 24-MC strain ST11064 was re-streaked from glycerol stocks stored at −80 °C onto a YPD agar plate and incubated at 30 °C for 48 h. The preculture was prepared by inoculating strain ST11064 biomass from the plate into 50 ml YPD medium in a 250 ml shake flask and incubating at 30 °C for 24 h with 250 r.p.m. agitation. Next, 5 ml of preculture was used to inoculate 115 ml of batch medium to a starting OD_600_ of 0.25. For Ambr 250 cultivation, the batch medium comprised mineral medium supplemented with yeast extract (10 g l^–1^) and citric acid (20 g l^–1^). The mineral medium was prepared with 0.5 g l^–1^ MgSO_4_⋅7H_2_O, 14.4 g l^–1^ KH_2_PO_4_, 0.1% (v/v) vitamin solution and 0.2% (v/v) trace metal solution as previously described^45^, but with 3.4 g l^–1^ NH_4_Cl and glycerol as the carbon source (40 g l^–1^).

The temperature was held constant at 30 °C. Dissolved oxygen was maintained above 20% by using a cascade of stirring speed ranging from 600 to 3,000 r.p.m. and aeration up to 1 volume air per volume growth medium per minute. The pH was maintained at 5.5 through the automatic addition of 1 M NaOH and 2.6 M H_3_PO_4_. Antifoam 204 (Sigma) was added automatically. Online measurements of acid and base addition, carbon dioxide evolution rate, dissolved oxygen and stirring speed were recorded for each reactor. The feed medium comprised 250 g l^–1^ glycerol. Feeding was automatically initiated once the carbon dioxide evolution rate dropped below 50% at the end of the batch phase. Feeding was set to a constant rate of 0.9 ml h^–1^. Samples were taken from each reactor every 6 h for the first 24 h and then every 12 h and immediately frozen until preparation for analyses. DCW and sterol content were determined from 1 ml samples as described above for small-scale cultivation.

For larger scale fermentation, strains were cultivated by fed-batch fermentation in a 5-litre bioreactor (BIOSTAT B-DCU, Sartorius). All fermentations were carried out in duplicate. For each of the strains W29, the TET strain ST11005 and the mixed-sterol strain ST12178, the strain was re-streaked from glycerol stocks onto a YPD agar plate and incubated at 30 °C for 24 h. The preculture was prepared by inoculating strain biomass from the plate into 50 ml YPD medium in a 250 ml shake flask and incubating at 30 °C for 24 h with 250 r.p.m. agitation. The volume of preculture required to inoculate a 2-litre batch medium to a starting OD_600_ of 2.5 was centrifuged for 10 min at 4,000*g* and concentrated to 10 ml volume. This cell suspension was used to inoculate the bioreactors. The bioreactors were equipped with pH, pO_2_ and temperature probes. The temperature was held constant at 30 °C. Dissolved oxygen was maintained above 20% by adjusting stirring between 600 and 1,200 r.p.m. and aeration (using a horseshoe sparger) between 0.5 and 3 standard-litre per min. The pH was kept at 5.5 through the automatic addition of 5 M NaOH. Antifoam A (Sigma) was added as required.

The batch medium comprised mineral medium supplemented with yeast extract (20 g l^–1^) and peptone (40 g l^–1^). The mineral medium was prepared with 0.5 g l^–1^ MgSO_4_⋅7H_2_O, 14.4 g l^–1^ KH_2_PO_4_, 0.1% (v/v) vitamin solution and 0.2% (v/v) trace metal solution as previously described^45^, 40 g l^–1^ glucose and 1 ml l^–1^ antifoam A (Sigma). The feed contained 5 g l^–1^ MgSO_4_⋅7H_2_O, 30 g l^–1^ KH_2_PO_4_, 1% (v/v) vitamin solution and 2% (v/v) trace metal solution as previously described^46^, with 300 g l^–1^ glucose. An exponential feeding profile was programmed, and feeding was initiated 24 h after inoculation. The feed rate, *F* (ml h^–1^), followed the profile *F* = 10 × e^(0.05 × *t*)^, where *t* is the time (h) from the start of feeding. After 36 h of exponential feeding, the feed was switched to a constant rate of 75 ml h^–1^ until the end of fermentation.

Duplicate samples from each reactor were taken every 8 h for the first 24 h and then every 12 h to measure DCW, sterol content, OD_600_ and glucose concentration. DCW and sterol content were determined from 1-ml samples as described above for small-scale cultivation. During fermentation, 1 ml of culture broth was centrifuged, and the supernatant was used to measure the glucose concentration using a glucose HK assay kit (Sigma). The supernatant was then filtered and frozen until further analyses. Glucose was later quantified using a Dionex Ultimate 3000 HPLC system equipped with a RI-101 refractive index detector (Dionex). An Aminex HPX-87H column (7.8 × 300 mm, Bio-Rad) with a Micro-Guard Cation H^+^ guard column (4.6 × 30 mm) heated to 30 °C was injected with a 10-µl sample. The mobile phase consisted of 5 mM H_2_SO_4_ with an isocratic flow rate of 0.6 ml min^–1^, which was held for 15 min. HPLC data were processed using Chromeleon software (v.7.2.9, Thermo Fisher Scientific). Glucose was identified and quantified using authentic standards. Glucose concentrations were calculated from the peak area by extrapolation from a six-point calibration curve regression.

### Honeybee diet preparation

The yeast strains W29, the TET strain ST11005 and the mixed-sterol strain ST12178 were cultivated using 5-litre fed-batch fermentation as described above. At the end of fermentation, the yeast biomass was recovered from the culture by centrifugation (4,000*g*, 20 min) and washed with deionized water. The biomass was heat-inactivated and dried (60 °C for a minimum of 24 h). The dried material was ground to a fine powder and stored at −20 °C until further use.

The yeast biomass cannot be subject to inactivation by autoclave or chemical treatment, as this will degrade the sterols present in the yeast. Incubation at 60 °C is commonly deemed sufficient to irreversibly inactivate yeast, and heat-inactivation of genetically modified yeast followed by feeding the inactivated yeast to live animals is a method that has been previously used in the United Kingdom^47^. Irreversible inactivation of the yeast was confirmed using a standard colony-forming unit assay. The heat-inactivated dried yeast was dissolved at 10 mg ml^–1^ in water. The suspension was plated in serial dilution (100 μl plated of 10, 1, 0.1, 0.01 and 0.001 mg ml^–1^ suspensions) on YPD agar and the plates were incubated at 30 °C for at least 7 days. No growth of *Y.* *lipolytica* colonies was observed. The detection limit is one organism per mg material or 10^6^ viable organisms per kg of material.

The yeast biomass was then incorporated into a meridic artificial diet at 20% w/w. Four diet types were prepared: a mixed-sterol yeast diet that contained the mixed-sterol strain ST12178; a wild-type yeast diet that contained strain W29; a platform yeast diet that contained the TET strain ST11005; and a base diet control without yeast supplementation. The base diet control was formulated to maintain total protein, sugar, sterol and fat content at the same level as in the yeast-supplemented diets. The content of this diet was a modified version of a previously described diet^48^. Specifically, the base diet contained 17% soy protein isolate (Soysol, MyVegan), 69.4% sugars (fructose, glucose, sucrose and maltodextrin), 6% lipids, 6.50% deionized water, 0.100% vitamin and mineral supplement (Latshaw Apiaries), 0.6% commercial phytosterol mix (BulkSupplements; Supplementary Table 7) and 0.400% carrageenan kappa. The diets were divided into 50 g patties and stored at −20 °C until use. The yeast-supplemented diets had the same proportion of protein (17%), carbohydrates (70%) and fats (6%) adjusted from the reagents of the base diet to accommodate nutrients present in the yeast. Specifically, the yeast-supplemented diets contained the following components: 20.0% dried yeast powder, 7.80% soy protein isolate (Soysol, MyVegan), 63.4% sugars, 0.1% commercial phytosterol mix (BulkSupplements, approximately 55% purity containing a mixture of sterols and stanols; Supplementary Table 7), 4% lipids, 4.20% deionized water, 0.100% vitamin and mineral supplement (Latshaw Apiaries) and 0.400% carrageenan kappa (Special Ingredients).

### Yeast-feeding trials

For the sterol analysis of honeybee brood, which is used as a proxy for the natural sterol profile of honeybee pupae, we sampled worker, drone and queen pupae from naturally fed colonies in our apiary (Buckfast queens, John Krebs Field Station, Oxford). Worker pupae were directly collected from capped brood frames. Drone pupae were collected from capped drone comb (larger cell size). Queen pupae were reared by grafting young larvae (2–3 days after hatching) into Nicot Queen Rearing Cups (Paynes Bee Farms). These were placed in queenless colonies in repurposed Styrofoam mini-nucleus hives (APIDEA) for up to 8 days until development to the capped brood stage. Tissues (3 pupae per replicate, *n* = 5) were sampled into preweighed 20 ml glass vials and the fresh weight was recorded before storing at −80 °C until further analysis.

Feeding trials were conducted using honeybee colonies maintained in repurposed Styrofoam mini-nucleus hives made up of one brood box with five frames and a top feeder with a hole for patty delivery. Hives were maintained in a closed glasshouse environment designed to prevent bee escape during the period between July and October 2022. Hives were distributed across two glasshouse rooms with varying entrance orientation. Feeders with 30% w/v sugar solution and water were distributed inside the glasshouse and replenished as required. The in-hive and ambient temperature and humidity were recorded every 30 min using autonomous in-hive sensors (Supplementary Data 3). A misting system was installed to cool the temperatures in the glasshouse.

Initially, each hive contained 900–1,200 adult bees, 2–3 frames of brood, larvae and eggs and 1–2 frames of honey stores, but no bee bread. Newly mated queens were introduced in cages with beekeeping candy (Candito, PIDA), for slow release, 3 days before the start of the experiment.

Feeding trials were conducted over 3 months from June to September 2022 at the John Krebs Field Station, Oxford. Six hives were randomly assigned to each treatment group (*n* = 6). At the start of the experiment, diet patties were added through the top feeder and replaced throughout the experiment as required. Hive weight (after removal of the diet patty) and patty weight were measured. The number of bee seams (one seam defined as a continuous line of bees between adjacent frames, observed after initial hive opening) and frames filled with honey (sugar stores) were estimated by visual inspection. The presence of the mated queen, sugar stores, eggs, larvae and capped brood were checked, and brood frames were photographed for subsequent counting. Eggs, larvae and capped brood were counted using the Adobe Photoshop count tool. Full assessments of the hives were conducted every 15 days.

Six days after each full assessment, hives were partially assessed with minimal disruption to the colony. Hive weight and patty weight were measured, and bee seams and sugar stores were estimated by visual inspection. The presence of the mated queen, eggs, larvae and capped brood were briefly checked. On days 21 and 45, hives with low populations (fewer than four bee seams) were topped up with orphanized nurse bees from mixed, naturally fed colonies.

At every assessment, nurse bee and pupae samples were taken from three hives from each treatment group. Six nurse bees and six pupae were collected from each of the sampled hives. Samples were collected into preweighed 20 ml glass scintillation vials. The fresh weights of the samples were measured, and the vials were stored at −80 °C. Nurse bees were dissected to separate the guts and gut contents from the rest of the tissues. This was done by partially thawing the samples on ice and pulling the guts from the abdomen by the stinger. The gut contents were transferred to a 2 ml microcentrifuge tube and the remaining tissues were returned to the 20 ml vial. Dissected samples were stored at −80 °C until further analysis.

### Pollen-starvation trial

A semi-field pollen starvation trial was conducted from August to October 2023 at the John Krebs Field Station, Oxford. Colonies were housed in mini-nucleus hives set up in an identical manner to the yeast-feeding trial and were maintained in one room of a mesh polytunnel purpose-built to prevent bee escape. One week before the start of the treatment, colonies were topped up with nurse bees from mixed, naturally fed colonies so that each box contained at least five bee seams and re-queened where necessary. We used a mix of pre-existing colonies and newly established colonies, but all were fed pollen for at least 1 month before the start of the experiment and were producing brood.

Buckets of water and feeders with 30% w/v sugar solution were distributed inside the polytunnel. Colonies were supplied with pollen or candy patties. Pollen patties consisted of 80% multifloral pollen and 20% high-concentrated sugar syrup (around 70% w/v). Candy patties consisted of about 80% beekeeping candy and around 20% maltodextrin, which was added to slow consumption and reduce melting of the patty in the hive. After 1 month of a feeding period, colonies in treatment groups 1, 2 and 3 were deprived of pollen for the corresponding number of weeks and fed with candy only. The control group (0) was fed pollen throughout. Treatment groups were balanced across hive entrance orientations, colony strengths (bee seams) and position in the polytunnel.

We performed a partial assessment every week to measure patty weight and hive weight and estimate bee seams. Every 2 weeks, a full assessment recorded the presence of the mated queen, eggs, larvae and capped cells, and the amount of sugar and pollen stores, and every frame was photographed. The photographs were used to count the number of cells with eggs, larvae and pupae in each hive using ImageJ^49^.

### Statistics and reproducibility

All graph plotting and statistical analyses were performed in R^50^ (except Extended Data Fig. 8, which was created in GraphPad Prism and analysed using SPSS). Data were tested for normality when appropriate. GLMs and GLMMs were fitted using stats^50^ and glmmTMB^51^ packages. Models with nonsignificant interaction terms were re-run without the interaction term. Post hoc analysis was performed using the car^52^ and emmeans^53^ packages with Tukey adjustments for family-wise error rates.

The mean relative abundance of each of the major sterols in naturally fed honeybee pupae was calculated as a percentage of the total sterol content. We compared sterol relative abundance across pupal types using a GLM with quasi-binomial distribution (relative abundance modeled as a function of (~) sterol type × pupal type). Sterol concentrations in pupae were calculated from the fresh weights of the pupal tissue. We compared sterol concentrations in pupal tissue across types using GLMs with Gaussian distributions (sterol concentration ~ sterol type × pupal type). We compared the relative abundance of sterols in pollen using a GLM with quasi-binomial distribution (relative abundance ~ sterol type). The coefficient of variation for each sterol was calculated by dividing the standard deviation by the mean relative abundance values of each sterol.

Counts for each brood type were compared across diet treatment groups using GLMMs, fitted to counts from day 45 onwards, with hive identifier (ID) as a random effect and negative binomial distributions (brood count ~ diet × time + (1|hive ID)). For egg counts, the interaction term was excluded (egg count ~ diet + time + (1|hive ID)).

Both the total diet provided to each colony and the total diet consumption by each colony were compared across diet treatment groups by fitting GLMs with Gaussian distribution (diet weight ~ diet). Because the hive weight correlated significantly with bee seams and the consumption rate correlated significantly with the hive weight for all treatment groups (Extended Data Fig. 8), the daily consumption rates in each interval were normalized by hive weight as a proxy for colony size. The normalized consumption rates were compared across diet treatment groups using a GLMM, with hive ID as a random effect and Gaussian distributions (normalized consumption rate ~ diet + time + (1|hive ID)).

The weight of each hive was compared across diet treatment groups using a GLMM, with hive ID as a random effect and Gaussian distributions (hive weight ~ diet + time + (1|hive ID)). The number of bee seams in each hive and the number of frames filled with honey were doubled to give integer values and compared across diet treatment groups using GLMMs, with hive ID as a random effect and Poisson distributions (2 × bee seams ~ diet + time + (1|hive ID); 2 × sugar stores ~ diet + time + (1|hive ID)).

For the sterol contents in the bodies and guts of nurse bees and of pupae (μg per individual), GLMMs were fitted for each sample type in each sterol, with Gaussian distributions and hive ID as a random effect (sterol content ~ diet × time + (1|hive ID)). Interaction terms were not significant in some models (total sterol in pupae, 24-MC in pupae, CAMP in the bodies and guts of nurse bees, ISOFUC in pupae, DESMO in pupae and guts of nurse bees) and were removed as appropriate.

We examined the relationships between variables measured during feeding trials by fitting GLMs (response variable ~ predictor variable × diet). All models used Gaussian distributions, apart from when comparing capped brood counts to the total sterol content of bodies of nurse bees from the same colony. In this case, a negative binomial distribution was used. When no significant interaction was found between diet treatment group and the predictor variable, the interaction term was excluded from the models (response variable ~ predictor variable + diet). For each diet treatment group, linear regressions were fitted between the predictor and response variables of interest.

All experiments in the study were performed once. Apart from bioreactor cultivations, a minimum of three biologically independent replicates were collected for all experiments. For bioreactor cultivations, two biological replicates with two technical replicates each were performed for measurement of all parameters. Replicates gave similar results for all experiments.

### Reporting summary

Further information on research design is available in the Nature Portfolio Reporting Summary linked to this article.

## Online content

Any methods, additional references, Nature Portfolio reporting summaries, source data, extended data, supplementary information, acknowledgements, peer review information; details of author contributions and competing interests; and statements of data and code availability are available at 10.1038/s41586-025-09431-y.

## Supplementary information

Supplementary Information Supplementary Tables 1–7 and supplementary references.

Reporting Summary

Supplementary Data 1 Subset of pollen sterol composition data from Zu et al. (2021) from bee-pollinated plants.

Supplementary Data 2 Statistical analysis on the sterol composition of samples taken from colonies used during the yeast-feeding trial.

Supplementary Data 3 Hive temperature and humidity recordings taken during the yeast-feeding trial.

## Source data

Source Data Fig. 1

Source Data Fig. 3

Source Data Fig. 4

Source Data Extended Data Fig. 2

Source Data Extended Data Fig. 3

Source Data Extended Data Fig. 4

Source Data Extended Data Fig. 5

Source Data Extended Data Fig. 6

Source Data Extended Data Fig. 9

## Data Availability

All data are available from Figshare (10.6084/m9.figshare.22820849)^54^. Source data are provided with this paper.
