# Supplementary material for: Engineered yeast provides rare but essential pollen sterols for honeybees
Source: Nature. 2025 Aug 20;646(8084):365–71. doi: 10.1038/s41586-025-09431-y (PMC12507675; doi:10.1038/s41586-025-09431-y)
Supplement: Supplementary file 1 — Supplementary Tables 1–7 and supplementary references. [file 41586_2025_9431_MOESM1_ESM.pdf]

nature portfolio

https://doi.org/10.1038/s41586-025-09431-y

Supplementary information

Engineered yeast provides rare but essential pollen sterols for honeybees

In the format provided by the authors and unedited

Supplementary Tables

Supplementary Table 1.

Yeasts engineered for non-native sterol production, in previous work and in this study. The highest production levels in each study are given, along with the carbon source used during the corresponding cultivation.

**Sterol**	**Host**	**Genotype *giving highest content only**	**Carbon source**	**Content/titre**	**Reference**
Epi-campesterol (‘ergosta-5-eneol’)	*S. cerevisiae* FY1679-28C (S288C derivative)	*AtDHCR7*	Glucose/ethanol/ galactose	45% w/w total sterol	55
Epi-campesterol (‘ergosta-5-eneol’)	*S. cerevisiae* FY1679-28C (S288C derivative)	*erg5Δ AtDHCR7*	Glucose/ethanol/ galactose	0.3 mg/l/OD (free sterol only)	56
Epi-campesterol	*S. cerevisiae* RH2881	*erg5ΔDrDHCR7*	Glucose (2% w/v)	98% w/w total free sterol Total sterol: 3.0 ug/10^7 cells	57
Epi-campesterol	*Y. lipolytica* ATCC201249	*erg5Δ XlDHCR7*	Sunflower seed oil (batch: 3.7% v/v, then maintained < 2g/L)	453 mg/L, inferred content: 20 mg/g DCW (5-L bioreactor, fed-batch)	58
Campesterol	*S. cerevisiae* BY4742	*erg4Δerg5ΔArDWF1* (plasmid)	Glucose (2% w/v) Galactose (2% w/v)	Not quantified	59
Epi-campesterol	*Y. lipolytica* ATCC201249	*erg5ΔDrDHCR7 ↑POX2*	Sunflower seed oil (batch: 28.86 g/L, then maintained < 2g/L)	942 mg/L, inferred content: 36 mg/g DCW (5-L bioreactor, fed-batch)	60
Epi-campesterol	*Y. lipolytica* PO1f (ATCC No. MYA-2613)	*erg5Δmfe1Δ XlDHCR7* (2x)	Glucose (batch: 40 g/L, maintained >10 g/L by feeding 50% glucose solution)	837 mg/L, inferred content: 32 mg/g DCW (5-L bioreactor, fed-batch)	61
Campesterol	*S. cerevisiae* CENPK2.1D	*AtDWF7 AtDWF5 AtDWF1 ↑ (ERG7 ERG8 ERG10 ERG12 ERG13 ERG19 ERG20 UPC2 tHMG1 (3x))*	Glucose (2% w/v)	41 mg/L	62
Epi-campesterol	*S. cerevisiae* YS5	*erg5ΔXlDHCR7*	Glucose (20 g/L)	178 mg/L (3 mL test tube)	63
Epi-campesterol	*S. cerevisiae* BY-T30 (BY4742)	*StDWF5 ↑ (ERG1 ERG8 ERG9 ERG10 ERG12*	Glucose (2% w/v)	7.8 mg/L/OD600 (shake flask)	64

		ERG13<br>*ERG19*<br>*ERG20*<br>*IDI1*<br>*tHMG1)*			
Cholesterol	*S. cerevisiae* RH2881	*erg5Δerg6Δ*<br>*DrDHCR7*<br>*DrDHCR24*	Glucose (2% w/v)<br>Molasses (0.75% v/v)	96% w/w total sterol,<br>1mg/g wet weight of yeast,<br>10 mg/L	57
Cholesterol	*P. pastoris CBS7435 Δhis4Δku70*	*erg5Δerg6ΔDrDHCR7*<br>*DrDHCR24*	Glycerol (1% w/v)<br>Methanol (1% w/v)	89% of total sterol (shake flask)	65
Cholesterol	*S. cerevisiae* BY-T3 (BY4742)	*erg6Δatf2Δ*<br>*DrDHCR7*<br>*DrDHCR24*<br>↑<br>*(ERG1*<br>*ERG9*<br>*ERG20)*	Glucose (2% w/v)<br>Galactose (2% w/v)	16 mg/L (250 mL shake flask)	66
Cholesterol	*S. cerevisiae* BY-T30 (BY4742)	*StDWF5 GgDHCR24*<br>↑<br>*(ERG1*<br>*ERG8*<br>*ERG9*<br>*ERG10*<br>*ERG12*<br>*ERG13*<br>*ERG19*<br>*ERG20*<br>*IDI1*<br>*tHMG1)*	Glucose (2% w/v)	8.5 mg/L/OD600 (shake flask)	64
Cholesterol (intermediate in diosgenin synthesis)	*S. cerevisiae* (BY4742)	*atf2Δ*<br>*StDWF5 GgDHCR24*<br>↓ERG6 *Vc*CYP90B27<br>*Dzin*CYP90G6<br>*Vc*CYP94N1<br>*Vv*CPR<br>*Vc*CYP90B27<br>*Vc*CYP94N1<br>*Vv*CPR<br>↑<br>*(ERG1*<br>*ERG8*<br>*ERG9*<br>*ERG10*<br>*ERG12*<br>*ERG13*<br>*ERG19*<br>*ERG20*<br>*IDI1*<br>*tHMG1)*	Glucose (2% w/v)<br>(Fed to 5 g/L)	170 mg/g DCW (shake flask),<br>5.5 g/L (5-L fermentation)	64
Desmosterol	*S. cerevisiae* RH2881	*erg6ΔDrDHCR7*	Glucose (2% w/v)	87% w/w total free sterol<br>Total sterol: 3.4 ug/10^7 cells	57
Desmosterol	*S. cerevisiae* BY4742	*erg6ΔStDWF5*	Glucose (2% w/v)<br>Galactose (2% w/v)	Not quantified	32
β-Sitosterol	*S. cerevisiae* CENPK2.1D	*erg4Δ*<br>*AtDWF7*<br>*AtDWF5*<br>*AtDWF1*<br>*SMT2 (plasmid)*		2 mg/L	62

		are1Δ<br>*are2Δ*<br>↑<br>*(ERG10*<br>*ERG12*<br>*ERG13*<br>*tHMG1)*			
7-Dehydrocholesterol	*S. cerevisiae* RH2881	*erg5Δerg6ΔDrDHCR24*	Glucose (2% w/v)	86% w/w total free sterol, Total sterol: 3.2 ug/10^7 cells	57
7-dehydrocholesterol	*S. cerevisiae* RH2881	*erg5Δerg6ΔGgDHCR24*<br>↑ *(ERG1*<br>*ERG2*<br>*ERG3*<br>*ERG11*<br>*Erg24*<br>*ERG25*<br>*ERG26*<br>*ERG27)*	Glucose (4% w/v)<br>Galactose (1% w/v)	360.6 mg/L (shake-flask)	38
24-Epi-ergosterol	*S. cerevisiae* CICC1746 (industrial ergosterol production strain)	*ArDWF1*<br>*erg4Δ*↑ *(ERG5, YEH1, YEH2, ARE2, UPC2)*	Glucose (batch: 10 g/L glucose, feed: 500 g/L)<br>Ethanol	2.76 g/L<br>19.27 mg/gDCW<br>(2-L bioreactor, fed-batch)	37
24-Methylenecholesterol	*S. cerevisiae* BY4742	*erg4Δerg5ΔStDWF5*	Glucose (2% w/v)<br>Galactose (2% w/v)	Not quantified	32
24-Methylenecholesterol	*S. cerevisiae* YS5	*erg4Δerg5ΔXlDHCR7 (2x)*	Glucose (20 g/L)	225 mg/L (250 mL shake flask)	63
24-Methylenecholesterol	*Y. lipolytica* ST9100 (W29 derivative)	*erg4Δ erg5Δ*<br>*SeACS TtSTC1*<br>*Tsp**DWF7*<br>↑<br>*(HMG1, ERG12, ACL1, IDI1, ERG20)*	Glucose (8% w/v)	42.2 mg/g DCW (24-deep well plate)	This study
Desmosterol + cholesterol	*S. cerevisiae* BY4742	*erg6ΔStDWF5*<br>*SlSSR2*	Glucose (2% w/v)<br>Galactose (2% w/v)	Not quantified	32
24-Methylenecholesterol + campesterol	*S. cerevisiae* BY4742	*erg4Δ*<br>*erg5ΔStDWF5*<br>*SlSSR1*	Glucose (2% w/v)<br>Galactose (2% w/v)	Not quantified	32
24-Methylenecholesterol + cholesterol + desmosterol (inferred)	*S. cerevisiae* BY4742	*erg4Δ*<br>*erg5ΔStDWF5*<br>*SlSSR2*	Glucose (2% w/v)<br>Galactose (2% w/v)	Not quantified	32
24-Methylenecholesterol + campesterol	*Y. lipolytica* ST9100 (W29 derivative)	*erg4Δ erg5Δ*<br>*SeACS TtSTC1*<br>*Tsp**DWF7*<br>*StSSR1*<br>↑<br>*(HMG1, ERG12, ACL1, IDI1, ERG20)*	Glucose (8% w/v)	20.3 mg/g DCW (24-deep well plate)	This study
24-Methylenecholesterol + campesterol + isofucosterol + sitosterol	*Y. lipolytica* ST9100 (W29 derivative)	*erg4Δ erg5Δ*<br>*SeACS TtSTC1*<br>*Tsp**DWF7*<br>*StSSR1*<br>*CqSMT2*<br>↑<br>*(HMG1, ERG12, ACL1, IDI1, ERG20)*	Glucose (8% w/v)	20.3 mg/g DCW (24-deep well plate)	This study

24-Methylenecholesterol + campesterol + isofucosterol + sitosterol + cholesterol + desmosterol	*Y. lipolytica* ST9100 (W29 derivative)	*erg*4Δ *erg*5Δ<br>*SeACS TtSTC1*<br>*T*sp*DWF7*<br>*StSSR1*<br>*CqSMT2*<br>*SlDHCR24*<br>↑<br>*(HMG1, ERG12, ACL1, IDI1, ERG20)*	Glucose (8% w/v) (Bioreactor batch media 40 g/L glucose, feed media 300 g/L glucose)	24.6 mg/g DCW (24-deep well plate)<br>9.11 mg/g DCW (5 L bioreactor)	This study

Supplementary Table 2.
Yarrowia lipolytica strains used in this study. Corresponding genotypes, parental strains and elements used for the construction of each strain are given.

			**Elements used to construct strain**	
**Strain name**	**Genotype**	**Parent strain/Reference**	**gRNA vector**	**Integration vector/BioBrick**
ST4842 'W29 strain'	MATa	*Y. lipolytica* W29 (MatA, ATCC©20460™) strain Y-63746 from the ARS culture collection		
ST6512	MATa ku70Δ::PrTEF1->Cas9- TTef12::PrGPD->DsdA-TLip2	ST4842, Marella *et al.* (2020)[67]		
ST9100 'Platform strain'	MATa ku70Δ::PrTEF1->Cas9- TTef12::PrGPD->DsdA-TLip2 IntC_2-HMG1<-PrGPD-PrTefInt- >ERG12 IntC_3-SeACS<-PrGPDPrTefInt->YlACL1 IntD_1-IDI1<-PrGPD-PrTefInt->ERG20	ST6512, Arnesen *et al.* (2020)[23]		
ST11005 'Tet strain'	MATa ku70Δ::PrTEF1-Cas9-TTef12::PrGPD-DsdA-TLip2 IntC_2-HMG1<-PrGPD-PrTefInt->ERG12 IntC_3-SeACS<-PrGPD-PrTefInt->YlACL1 IntD_1-IDI1<-PrGPD-PrTefInt->ERG20 IntE_3-PrGPAT->TtSTC	ST9100	pCfB6637 (pNat-YLgRNA3_IntE_3)	pCfB10494 (IntE3_PrGPAT->TtSTC)
ST11014	MATa ku70Δ::PrTEF1-Cas9-TTef12::PrGPD-DsdA-TLip2 IntC_2-HMG1<-PrGPD-PrTefInt->ERG12 IntC_3-SeACS<-PrGPD-PrTefInt->YlACL1 IntD_1-IDI1<-PrGPD-PrTefInt->ERG20 IntE_3-PrGPAT->TtSTC Δerg5_HphMX	ST11005		BB5098 (Erg5_HphMX_KO)
ST11027	MATa ku70Δ::PrTEF1-Cas9-TTef12::PrGPD-DsdA-TLip2 IntC_2-HMG1<-PrGPD-PrTefInt->ERG12 IntC_3-SeACS<-PrGPD-PrTefInt->YlACL1 IntD_1-IDI1<-PrGPD-PrTefInt->ERG20 IntE_3-PrGPAT->TtSTC erg5Δ	ST11014		pCfB6611 (pNat-PrExp-Cre)
ST11040	MATa ku70Δ::PrTEF1-Cas9-TTef12::PrGPD-DsdA-TLip2 IntC_2-HMG1<-PrGPD-PrTefInt->ERG12 IntC_3-SeACS<-PrGPD-PrTefInt->YlACL1 IntD_1-IDI1<-PrGPD-PrTefInt->ERG20 IntE_3-PrGPAT->TtSTC erg5Δ erg4Δ_HphMX	ST11027		BB5097 (Erg4_HphMX_KO)
ST11056	MATa ku70Δ::PrTEF1-Cas9-TTef12::PrGPD-DsdA-TLip2 IntC_2-HMG1<-PrGPD-PrTefInt->ERG12 IntC_3-SeACS<-PrGPD-PrTefInt->YlACL1 IntD_1-IDI1<-PrGPD-PrTefInt->ERG20 IntE_3-PrGPAT->TtSTC erg5Δ erg4Δ_HphMX IntE_4-PrTEFInt->DHCR7_Stuberosum	ST11040	pCfB6638 (pNat-YLgRNA2_IntE_4)	pCfB10249 (IntE4_PrTEFInt->DHCR7_Stuberosum)
ST11057	MATa ku70Δ::PrTEF1-Cas9-TTef12::PrGPD-DsdA-TLip2 IntC_2-HMG1<-PrGPD-PrTefInt->ERG12 IntC_3-SeACS<-PrGPD-PrTefInt->YlACL1 IntD_1-IDI1<-PrGPD-PrTefInt->ERG20 IntE_3-PrGPAT->TtSTC erg5Δ erg4Δ_HphMX IntE_4-PrTEFInt->DHCR7_Drerio	ST11040	pCfB6638 (pNat-YLgRNA2_IntE_4)	pCfB10250 (IntE4_PrTEFInt->DHCR7_Drerio)
ST11058	MATa ku70Δ::PrTEF1-Cas9-TTef12::PrGPD-DsdA-TLip2 IntC_2-HMG1<-PrGPD-PrTefInt->ERG12 IntC_3-SeACS<-PrGPD-PrTefInt->YlACL1 IntD_1-IDI1<-PrGPD-PrTefInt->ERG20 IntE_3-PrGPAT->TtSTC erg5Δ erg4Δ_HphMX IntE_4-PrTEFInt->DHCR7_Ldrancou	ST11040	pCfB6638 (pNat-YLgRNA2_IntE_4)	pCfB10251 (IntE4_PrTEFInt->DHCR7_Ldrancou)
ST11059	MATa ku70Δ::PrTEF1-Cas9-TTef12::PrGPD-DsdA-TLip2 IntC_2-HMG1<-PrGPD-PrTefInt->ERG12 IntC_3-SeACS<-PrGPD-PrTefInt->YlACL1 IntD_1-IDI1<-PrGPD-PrTefInt->ERG20 IntE_3-PrGPAT->TtSTC erg5Δ erg4Δ_HphMX IntE_4-PrTEFInt->DHCR7_Esilicul	ST11040	pCfB6638 (pNat-YLgRNA2_IntE_4)	pCfB10252 (IntE4_PrTEFInt->DHCR7_Esilicul)
ST11060	MATa ku70Δ::PrTEF1-Cas9-TTef12::PrGPD-DsdA-TLip2 IntC_2-HMG1<-PrGPD-PrTefInt->ERG12 IntC_3-SeACS<-PrGPD-PrTefInt->YlACL1 IntD_1-IDI1<-PrGPD-PrTefInt->ERG20 IntE_3-PrGPAT->TtSTC erg5Δ erg4Δ_HphMX IntE_4-PrTEFInt->DHCR7_Cprotoch	ST11040	pCfB6638 (pNat-YLgRNA2_IntE_4)	pCfB10253 (IntE4_PrTEF->DHCR7_Cprotoch)

ST11061	MATa ku70Δ::PrTEF1-Cas9-TTef12::PrGPD-DsdA-TLip2 IntC_2-HMG1<-PrGPD-PrTefInt->ERG12 IntC_3-SeACS<-PrGPD-PrTefInt->YlACL1 IntD_1-IDI1<-PrGPD-PrTefInt->ERG20 IntE_3-PrGPAT->TtSTC erg5Δ erg4Δ_HphMX IntE_4-PrTEFInt->DHCR7_Csubellip	ST11040	pCfB6638 (pNat-YLgRNA2_IntE_4)	pCfB10254 (IntE4_PrTEFInt->DHCR7_Csubellip)
ST11062	MATa ku70Δ::PrTEF1-Cas9-TTef12::PrGPD-DsdA-TLip2 IntC_2-HMG1<-PrGPD-PrTefInt->ERG12 IntC_3-SeACS<-PrGPD-PrTefInt->YlACL1 IntD_1-IDI1<-PrGPD-PrTefInt->ERG20 IntE_3-PrGPAT->TtSTC erg5Δ erg4Δ_HphMX IntE_4-PrTEFInt->DHCR7_Mverticillata	ST11040	pCfB6638 (pNat-YLgRNA2_IntE_4)	pCfB10255 (IntE4_PrTEFInt->DHCR7_Mverticillata)
ST11063	MATa ku70Δ::PrTEF1-Cas9-TTef12::PrGPD-DsdA-TLip2 IntC_2-HMG1<-PrGPD-PrTefInt->ERG12 IntC_3-SeACS<-PrGPD-PrTefInt->YlACL1 IntD_1-IDI1<-PrGPD-PrTefInt->ERG20 IntE_3-PrGPAT->TtSTC erg5Δ erg4Δ_HphMX IntE_4-PrTEFInt->DHCR7_Gsoja	ST11040	pCfB6638 (pNat-YLgRNA2_IntE_4)	pCfB10256 (IntE4_PrTEFInt->DHCR7_Gsoja)
ST11064 ’24-MC strain’	MATa ku70Δ::PrTEF1-Cas9-TTef12::PrGPD-DsdA-TLip2 IntC_2-HMG1<-PrGPD-PrTefInt->ERG12 IntC_3-SeACS<-PrGPD-PrTefInt->YlACL1 IntD_1-IDI1<-PrGPD-PrTefInt->ERG20 IntE_3-PrGPAT->TtSTC erg5Δ erg4Δ_HphMX IntE_4-PrTEFInt->DHCR7_Tsp	ST11040	pCfB6638 (pNat-YLgRNA2_IntE_4)	pCfB10257 (IntE4_PrTEFInt->DHCR7_Tsp)
ST11065	MATa ku70Δ::PrTEF1-Cas9-TTef12::PrGPD-DsdA-TLip2 IntC_2-HMG1<-PrGPD-PrTefInt->ERG12 IntC_3-SeACS<-PrGPD-PrTefInt->YlACL1 IntD_1-IDI1<-PrGPD-PrTefInt->ERG20 IntE_3-PrGPAT->TtSTC erg5Δ erg4Δ_HphMX IntE_4-PrTEFInt->DHCR7_Wchondrophi	ST11040	pCfB6638 (pNat-YLgRNA2_IntE_4)	pCfB10258 (IntE4_PrTEFInt->DHCR7_Wchondrophi)
ST11943 ‘Two-sterol strain’	MATa ku70Δ::PrTEF1-Cas9-TTef12::PrGPD-DsdA-TLip2 IntC_2-HMG1<-PrGPD-PrTefInt->ERG12 IntC_3-SeACS<-PrGPD-PrTefInt->YlACL1 IntD_1-IDI1<-PrGPD-PrTefInt->ERG20 IntE_3-PrGPAT->TtSTC erg5Δ erg4Δ_HphMX IntE_4-PrTEFInt->DHCR7_Tsp IntF3_PrDGA1->StSSR1	ST11064	pBP8003 (pNat-YLgRNA4-IntF_3)	pCfB10948 (IntF3_PrDGA1->StSSR1)
ST12140 ‘Four-sterol strain’	MATa ku70Δ::PrTEF1-Cas9-TTef12::PrGPD-DsdA-TLip2 IntC_2-HMG1<-PrGPD-PrTefInt->ERG12 IntC_3-SeACS<-PrGPD-PrTefInt->YlACL1 IntD_1-IDI1<-PrGPD-PrTefInt->ERG20 IntE_3-PrGPAT->TtSTC erg5Δ erg4Δ_HphMX IntE_4-PrTEFInt->DHCR7_Tsp IntF3_PrDGA1->StSSR1 IntE1_PrGPAT->SMT2_Cq	ST11943	pCfB6633 (pNat-YLgRNA2_IntE_1)	pCfB10851 (IntE1_PrGPAT->SMT2_Cquinoa)
ST12178 ‘Mixed-sterol strain’	MATa ku70Δ::PrTEF1-Cas9-TTef12::PrGPD-DsdA-TLip2 IntC_2-HMG1<-PrGPD-PrTefInt->ERG12 IntC_3-SeACS<-PrGPD-PrTefInt->YlACL1 IntD_1-IDI1<-PrGPD-PrTefInt->ERG20 IntE_3-PrGPAT->TtSTC erg5Δ erg4Δ_HphMX IntE_4-PrTEFInt->DHCR7_Tsp IntF3_PrDGA1->StSSR1 IntE1_PrGPAT->SMT2_Cq IntE5_PrGPAT->DHCR24_Sl	ST12140	pCfB10783 (pNatYlgRNA-IntE_5)	pCfB10853 (IntE5_PrGPAT->DHCR24_Slycopersic)

Supplementary Table 3.
Codon-optimised sequences of heterologous genes used for strain engineering in this study.

Gene	Nucleic acid sequence, codon optimised for *Yarrowia lipolytica*
Solanum tuberosum delta-7 sterol reductase	ATGGCCGAGTCTCAGCTGGTGCACCCTCCTCTGTTCACCTACATCTCTATGCTGGCCCTGCTGACCCTG<br>GTGCCTCCTTTCGTGATCCTGATGTGGTACACCAACGTGCACGCCGACGGCTCTGTGCTGCAGACCTT<br>CAACTACCTGAAGGAAAACGGCCTGCAGGGCCTGATCGACATCTGGCCCCGACCTACCGCCATTGCC<br>GGAAAGATCATCATCTGCTACGCCCTGTTCGAGGCTACCCTGCAGCTGCTGCTGCCCGGCAAGCGAGT<br>GCAGGGCCCCATCTCTCCCACCGGCCACCGACCTGTGTACAAGGCCAACGGCATGGCCGCCTACACC<br>GTGACTCTGATTACCTACCTGTCTCTGTGGTGGTTCGGCATCTTCAACCCCACCGTGGTGTACGACCA<br>CCTGGGCGAGATCCTGTCTACCCTGAACTTCGGCTCTCTGATCTTCTGCCTGTTCCTGTACATCAAGGG<br>ACACGTGGCTCCCTCTTCTACCGACCACGGCTCCTCTGGCAACATCATCGTGGACTACTACTGGGGCA<br>TGGAACTGTACCCTCGAATCGGCAAGCACTTCGACATCAAGGTGTTCACCAACTGTCGATTCGGCATG<br>GTGTCTTGGGGACTGCTGCCCATCACCTACTGCATCAAGCAGTACGAGGAATACGGATCTCTGTCTGA<br>CTCCATGCTGATCCACGCCATCATCACCCTGGTCTACGTGACCAAGTTCTTCTGGTGGGAGGCCGGCT<br>ACTGGAACACCATGGACATTGCCCACGACCGAGCCGGCTTCTACATCTGCTGGGGCTGCCTGGTGTTC<br>CTGCCTTGCATGTACACTTCTCCCGGCATGTACCTGGTGAAGCACCCCGTGAACCTGGGACCTCAGCT<br>GGCCATCTCCATCCTGGTGGCCGGCATCCTGTGCGTGTACATTAACTACGACTGCGACCGACAGCGAC<br>AAGAGTTCCGACGAACTAACGGCAAGGCCCTGGTGTGGGGCAAGGCTCCCTCCAAGATCGTGGCCTC<br>TTACACCACCACCACTGGCGAGACTAAGTCCTCTCTGCTGCTGACCTCCGGCTGGTGGGGCCTGTCTC<br>GACACTTCCACTACGTGCCCGAGATTCTGGCCTCTTTCTTTTGGTCTGTGCCCGCTCTGTTCAACCACA<br>TTATGCCCTACTTCTACGTGATCTACCTGACCGGCCTGCTGCTGGACCGAGCCAAGCGAGATGACGAA<br>CGATGCAAGTCTAAGTACGGCAAGTACTGGAAGAAGTACTGCGAGAAGGTCCCCTACCGAGTGATCC<br>CCGGCATCTACTAA
Danio rerio delta-7 sterol reductase	ATGATGGCCTCTGACCGAGTTCGAAAGCGACACAAGGGCTCTGCTAACGGTGCTCAGACCGTTGAGA<br>AGGAACCCTCCAAGGAGCCCGCCCAGTGGGGCCGAGCCTGGGAGGTCGATTGGTTCTCCCTGTCCGG<br>CGTGATTCTGCTGCTGTGCTTTGCCCCTTTCCTGGTCTTCTTCTTCATCATGGCTTGTGATCAGTACCAG<br>TGCTCTATCTCCCATCCCCTTCTGGACCTTTACAACGGTGACGCCACTCTGTTCACCATCTGGAACCGA<br>GCCCCCTCCTTCACCTGGGCCGCTGCCAAGATCTACGCCATCTGGGTCACCTTCCAGGTCGTTCTGTA<br>CATGTGCGTCCCCGACTTCCTGCACAAGATCCTGCCAGGTTACGTCGGCGGTGTCCAGGACGGAGCTA<br>GAACTCCCGCCGGCCTGATCAACAAGTATGAGGTTAACGGTCTGCAGTGCTGGCTCATCACCCACGT<br>GCTCTGGGTGCTGAATGCTCAGCACTTCCACTGGTTTTCACCCACCATTATCATTGACAACTGGATCC<br>CCCTGCTGTGGTGCACCAACATTCTGGGCTATGCCGTCTCCACCTTCGCTTTCATCAAGGCCTACCTGT<br>TCCCCACCAATCCCGAGGACTGCAAGTTCACCGGAAACATGTTTTACAATTACATGATGGGTATTGAG<br>TTCAACCCCCGAATCGGTAAGTGGTTCGACTTCAAGCTGTTCTTCAACGGTCGGCCTGGCATCGTCGC<br>CTGGACCCTCATCAACCTTTCCTACGCTGCTAAGCAGCAGGAGCTGTACGGCTACGTCACCAACTCTA<br>TGATCCTGGTCAACGTCCTGCAGGCCGTGTACGTTGTCGACTTCTTCTGGAACGAGGCTTGGTACCTG<br>AAAACCATCGACATCTGCCACGACCACTTTGGCTGGTACCTGGGATGGGGAGACTGCGTTTGGCTGC<br>CTTTCCTGTACACCCTGCAGGGTCTGTACCTGGTCTACAACCCTATCCAGCTGTCCACTCCCCACGCTG<br>CCGGCGTGCTGATCCTGGGTCTGGTCGGTTACTACATTTTTCGAGTGACCAACCACCAGAAGGACCTC<br>TTCCGACGAACTGAGGGCAACTGTTCGATCTGGGGCAAGAAGCCGACCTTTATCGAGTGCTCCTACC<br>GATCTGCCGACGGCGCCATCCACAAGTCCAAGCTTATGACCTCCGGCTTTTGGGGTGTTGCCCGACAC<br>ATGAACTACACTGGTGACCTTATGGGTTCCCTGGCCTACTGTCTGGCCTGTGGTGGTAACCACCTCCT<br>CCCCTACTTCTACATTGTCTACATGACTATTCTGCTGGTCCACAGATGCATTCGAGACGAGCACCGAT<br>GCTCCAACAAGTACGGCAAGGATTGGGAACGATACACCGCCGCCGTCTCTTACCGACTGCTGCCCAA<br>CATCTTCTAA
Legionella drancourtii delta-7 sterol reductase	ATGTACTTCAAGATCCGAAACACCCTGGGACCTCTGCTGCTGATCCTGTCTTGCCCCATCTTCGTGAT<br>GCTGATGTGGTACACCAACACCGAGCTGAAGGGATCTCTGTCTACCCTGTGGGACCTGATCGTGCAG<br>CAGGGCCTGTTCGAGACTACCTACAAGATCTGGCAGCCCTACTTCTGGGGCTCTGCCCTGGCCTGGAA<br>GGTGATCTTCGCCTTCATCATCTTCGAGCTGGCCCTGATGCGACTGCTGCCCGGCAAGGAATTCACCG<br>GACCTGTGACTCCCAAGGGCAACGTGCCCATCTACAAGGAAAACGGACCCCTGGCCTTCATTACCAC<br>CATGACTACCTTTTGCGTGGCCTCTTTCGGCCTGCATCTGTTCCCCGCCTCTATCCTGTACGACAACCT<br>GGGCGCCATCCTGGGAGCCCTGAACGTGTTCTCTCTGATCTTCTGCGCCCTGCTGTATATCAAGGGCC<br>GATACTTCCCCTCTTCTACCGACTCTGGCATCACCAACAACATCATTTTCGACTACTACTGGGGAACC<br>GAGCTGTACCCTCACATCTTCGGATGGTCTATCAAGAAGTTCATCACCTGTCGATTCGGCATGATGTC<br>TTGGGGACTGTTCCTGATCTCTTACTGCGCCAAGCAGGCCGAGCTGGGCGACCTGGCCAACTCTATGC<br>TGATCTCTGTGGCCCTGCAGTTCCTGTACCTGTCTAAGTTTTACCTGTGGGAGAAGGGCTACCTGCGA<br>TCTCTGGACATTATGCACGACCGAGCCGGCTTCTACATCTGCTGGGGCTGCCTGGTGTGGGTGCCCTG<br>CATCTACACTTCTCCCTCTATGTACCTGGTGCTGCACCCCATCCACCTGTCTTTCTGGCTGGCCACCTC<br>CATTCTGGTGCTGGGAGCCGCTTCCATCCTGATCAACTACTTCGCCGACCGACAGCGACTGATGACCC<br>GAGCCACCGACGGCGAGTGTAAGATCTGGGGAAAGAAGCCCGTCACCGTTTTCGCCCAGTACCAGAC<br>CACCGAGGGCGACAAGAAGCAGACCATCCTGCTGGCCTCTGGCTGGTGGGGCGTCGCCCGACACTTC<br>CACTACGTGCCCGAGCTGGCTGGAACCTTCTTCTGGTCTGTGCCCGCTCTGTTTGAGAACTTCTCTCCT<br>TACTTCTACCTGTGCTTCCTGACCATTCTGCTCGTGGACCGAGCCTTCCGAGATGACCGACGATGCTCT

	GACAAGTACGGACAGTACTGGCACAAGTACTGCGAGCTGGTGCCTTACAAGATCGTGCCCTTCGTGA<br>TCTAA
Ectocarpus siliculosus delta-7 sterol reductase	ATGATCGACGGCGCTGCCATCGGACGATCTCCCGTGATCTCTTCTTGGCACGGCTACAACCCCGCCTC<br>TTCGCAGCAGCAGTGTCTGCAGGCCGTCCAGACCTCTCTGCCCACCACCGACGGCGACCGAGAGCGA<br>CGACGATCTATGGCCCTGCGAACCTCTACCAAGCAGGCCTCTGACGCCATGGACATCCGAGAGGCCG<br>TGAAGGCCTCTGTGGCCGCCGAGTCTAAGGACTCTAAGCTGTGGGGCTTCGTGCCCAACTGGTTCCGA<br>ACTACCGTGGGACCCCTGTTCCTGATCCTGGTGCCTCCTTTCTTCGTGGTGTTCTGGTGGCACCTCCTG<br>GTGTCTCACTCTGGCTCTTGGGTGTCTCTGTGGGGCGACCTGAAGGCCGCTGGACCCGAGTACGTGCT<br>GGACGTGGTGCCCTCTCCTGTGGACCCCGCTGCCTGGAAGTACATCCTCGGCTTCGGCGTGTTCGAGA<br>TCCTGCTGATGGTGGGACTGCCCGGCAAGGCCTTCCGAGCTAACCCCACCGCCACCGGACACATCCC<br>CGTGTACAAGGCCAACGGCATGCTGTCTTACCTGGTGACCCTGGCTACCCTGTGCGCCCTGGTCGCCA<br>CCGACCGACTGGACCCCAAGAACGTGTACGACAAGCTGGGCGAGATCTTCACCGGCCTGTCTGTGTT<br>CTCTCTGTTCTTCGTGCTGCTGCTGACCGTGAAGGGCCTGTACTTCCCCTCTACCGACGACTCTGGATC<br>TAACGGATCTTTCCTGCAGAACTACTGGTGGGGCACCGAGCTGTACCCTCGAGTGTTCGGAGCCGAC<br>GTGAAGATGTTCACCAACTGCCGATTCGGCATGATGTACTGGGCCGTGGGCGCCGTGATCTACGCTTA<br>CACCCAGCAGCAGATGTACGGAAAGCTGTCCTCTTCTATGGCCGTGTCTGTGATCCTGCAGCTGACCT<br>ACATCACCAAGTTCTTCCACTGGGAGATGGGCTACATGAACTCTATGGACATTCAGCACGACCGAGC<br>CGGCTACTACCTGTGCTGGGGCTGCCTGGTGTGGGTGCCCGCCGTGTACTCTTCTCCCGGCATCTACC<br>TGGTCAAGCACCCCATTTCTCTCGGCTGGTACGGCGCCTCTGCCATTCTGGCCCTGGGCCTGCTGTCTA<br>TCTGGGCCAACTTCGACGCTGACCGACAGCGACACGCCTTCCGACAGGCCAAGGGCGACATCATCGT<br>GTGGGGCAAGCCCGCCAAGTACATCACCGCCGGCTACATCAACGCCCGAGGCGAGAAGGCCTCCTCT<br>CTGCTGCTCTGCACCGGCTGGTGGGGAGTCGCCCGACACTTCCACTACCTGCCTGAGATTACCGGCGC<br>CTTCTTCTGGACCGTGCCTGCTCTGTTCGAGACTCCCACTCCTTACTTCTACGTCGTGTTCCTGGTGCT<br>GCTCCTCACTGACCGAGCTTTCCGAGATGACACCCGATGCCGAGGCAAGTACGGCAAGCACTGGGAC<br>AAGTACTGCGCTCAGGTGCCCTACAAGATCGTGCCCGGCATCCTGTAA
Candidatus *Protochlamydia amoebophila* delta-7 sterol reductase	ATGCTGATCGAGATGCTGTGCATCACCAAGCACATCCTCGAGAAGATCTCTAAGTTCTCTCTGGCTCG<br>ACCCCACGTGGCCAACACCAACATTTTCTTCAGACAGACCTTCGGACCCCTGTTCCTGCTGCTGCTGT<br>GCCCTCCTACCGTGTTCGCCTTCTGGTACACCAACACCTACCTCGAGGGATCTCTGTTCCGATTCACCG<br>AGTTCGCCTGGCAGCAGGGCTTCCTGTCTACCCTCAAGACTATCTGGTTCCCCTACTTCTTCGGCACCT<br>CTATCGCCTGGACCATGCTGGCCATCTTCGCTTCTCTGCAGCTGATCCTGATGCGAATTCTGCCCGGC<br>GAGTGCTACGAGGGCCCCATCACTCCCACCGGACACGTGCCCCTGTACAAGGCCAACCGATTCTCTG<br>CCTTCATCGTGACCGTGTCTATCTTCCTGATCGCCTCTTGCTACTACCAGCTGTTCGCTCCCACCATCA<br>TCTACGACAACTTCCCCGGCCTGCTGGGCGCCCTGAACATCTTCTCCCTGTGTTTCTGCTTCTTCCTGG<br>TGCTGAAGGGCCACTACTTCCCTTCTAACGGCGACGTCGGCGGCTCTGGCAACATCATCTTCGACTAC<br>TACTGGGGCATGGAACTGTACCCTCGACTGCTCGGCTGGGACATCAAGCAGTTCACCAACTGCCGATT<br>CGGCATGATGTCTTGGGCCCTGATCGTGATCTCTTTCGCCGCCAAGCAGCAACAGCTGGACGGCCTGT<br>CTGACTCTATGTTCGTGGCCGTGGCTCTCCAGCTCATCTACATTACCAAGTTCTTCATCTGGGAGCCCG<br>GCTACCTGCGATCTCTGGACATTATGCACGACCGAGCCGGCTACTACATCTGCTGGGGCTGCCTGGTG<br>TGGGTGCCCGGCATCTACACTTCTCCCACTCTGTACCTGGTGGATCACCCCAACCACCTGGGACTCGC<br>CCTGTCCTCTCTGCTGTTCGTGGTGGGCGTGATCGGCATCCTGGTGAACTACCTGGCCGACCGACAGC<br>GACAGCTCGTGCGAAAGAACCAGGGCAACTGTCGAATCTGGGGAAAGGAACCCATCCTGACCATTGC<br>CAAGTACACCACTCAGACCGGCGAGACTAAGCAGAACCTGCTGCTCGCCTCTGGCTGGTGGGGCCTG<br>TCTCGACACTTCCACTACCTGCCTGAGCTGCTGGGAGCCTTCTGCTGGTCTGCTCCCGCTCTGTTCGAG<br>AACTTCCTGCCTTACTTCTACTTCGTGTTTCTGACCCTGCTCCTGACCGACCGAGCTTTCCGAGATGAC<br>CAGAGATGCTCTAAGAAGTACGGCGAGGACTGGAAGATCTACTGCCAGCGAGTGCCCTACAAGATCA<br>TCCCCTTCGTGATCTAA
Coccomyxa subellipsoidea delta-7 sterol reductase	ATGGTCACAACCCGAGCCGCTGCTCGAGCTCAGACCCCTCTGGGCAAAGCTCCTCCCTCCGACCTGTC<br>CCACTCCGAACCCTCCGTCTCTACTCAGAACGGCAAGAAGACATGGGCCGAGACCGCTGGGGCAGGC<br>GAGCACATCGGCGCCTGGGGTATCGGCGGCACTGCCGGACACGTCTTGGCCTACCTGGGTACTCTTGC<br>CCTTATGATCGGTTGCCCCGCCTTTGCAATCTACATGTGGTTTACCCTCACCCACTTGGATGGCTCTCT<br>TGTGGAGCTCGTCCAGTTCGCCCAGAAGGCTGGATTTCAGGGCGTCCGAGCCTCATGGCCCTGGCCCT<br>CCCAGGAGGCCTGGGCCATCATCGCCTCCTTCGGCGGTCTTCAGGCCTTCCTGCAGCTTGCCTTGCCC<br>GGTGCTGTGCACAAGGGCCCAGTCTCTCCCAAGGGTAACGTCCCCGTGTACAAGGCCAACGGTGTCC<br>TCGCCTACTTTACCACCCTGGCTCTGTTCGTCCTTGGCTGGCAGTTCAAGCTGTTTTCCCCCGCCAGAG<br>TCTACGACCTGTTCGGCGAGATCCTTTCCGGCCTGAACATGTTCTCTCTGCTGTTCTGCCTGTTCCTGT<br>ACTTCAAGGGCAAGTACGCCCCCTCATCCTCGGATTCTGGTTCTACCGGCTCCCTGATGTACGATTAT<br>TACTGGGGGATGGAGCTGTACCCTCGGATTGGACGACACTTTGACCTGAAGACTTGGACTAACTGCC<br>GAATGGGCATGATGGGCTGGGGAGTTCTGGTCCTGTGTTACGCTGTGAAGCAGCACGAGCTGTACGG<br>TTACCTTTCTAATTCGATGGCTGTTTCTATCCTGCTCATGCATTTATACATTTTCAAGTTCTTCCTCTGG<br>GAGACTGGTTACTGGGGTACCATGGACATCGCTCACGACCGCGCTGGATACTACCTCTGCTGGGGAT<br>GCCTGAACTGGGTCCCCGCTATCTACACCTCTCCCGCCCTGTACCTCGTCGAGAACCCTATTCAGTGG<br>TCTCTGCCCGCCGCCACCGCTATCGCTGTTGCCGGTACCCTGGCTATCTACATCAACTACGACTCCGA<br>TCGGCAGCGACAGGTTTTCCGAGCTACCAATGGTAAGGCCCTGGTGTGGGGCAAGCCTCCCCAAATT<br>ATCTCTGCCAAGTACATCACCGGCGATGGCAAGCAGAAGACCTCCCTGCTCCTGGCCTCTGGCTGGTG<br>GGGGCTCGCCCGACACTTCCACTACCTGCCCGAGATCTTGGCCGCCTTCTTTTGGACCCTCCCCGCTG

	GTATCTCCCACGCTCTGCCCTACTTTTACGTCTTCTTCCTGACTCTGCTCCTTACCGATCGAGCTTTCCG<br>AGACGACGTCCGATGTAGCTCCAAATACGGTGCCTACTGGCAGCAGTACACCAAGGCCGTTCCCTAC<br>AAGATGATCCCTTACATTTTCTAA
Mortierella verticillate delta-7 sterol reductase	ATGGCCGTGCAGCAGCGAAAGACCCCTGCTCAGGTGGACGTGAAGGCCGAGTCTAAGCTGGACGCCC<br>AGGTGGGCAAGACCTGGGGCCGAGATCGAGATGTGTCTTTCGGCACCATCCTGATCTCCCTGGGCAT<br>CCTGGTGATGTCTCCCATCTGGGTGATGTACACCTACATCTCTTGCAACGCCTACCAGTGCGCCATGT<br>CTGCTCCCGCTCTCGAGATCTACAACTCTCCCGACACTCTGGCCGCCATTCAGACCCTGCTGCTGCGA<br>GAGGTGCCCCGATTCTCTCCCTACGCCGCTCGACTGTTCTTCACCTGGCTGGCCTTCCAGGCCGCTCTG<br>TACGCCTTCCTGCCTGCTCAGATCGGCTACGGCCAGCGAACCCCTGCCGGCCACATTCTGCCCTACAA<br>GGTGAACGGCCTGCTGGCCTGGTTCATCTCTCACTCTATCTACGCTGCCGGCGGACTGTACTTCGGCT<br>GGTGGAAGCTGTCTATCATCCACGACAACTGGGGCGGACTGCTGGTGGCCGCCAACATGTACGGCTA<br>CTTTCTGACATTCTTCTGCTTTATCAAGGCCTACACCTTTCCTTCTCACCCCGCCGACCGAAAGTTCTC<br>TGGCTCTTTCATCTACGACCTGCTGATGGGCATCGAGTTCAACCCTCGAATCGGCAAGCTGTTCGACT<br>TCAAGCTGTTTCACAACGGACGACCCGGCATCGTGGCCTGGACCATGATCAACCTGTCCTTCGCCGCT<br>GCTCAGTACGAGAAGATCGGATACGTGACCAACTCTATGATCCTGCTGAACCTGCTGCATGCTACCTA<br>CGTGCTGGACTTCTTCTACAACGAGGACTGGTATCTGCGAACCATCGACATTGCCCACGACCACTTCG<br>GCTTCTACCTGGCCTGGGGCGACTCCGTGTGGCTGCCCTGGCTGTACACCCTGCAGTCTCACTACCTG<br>GTGCGAAACCCCGTGGACCTGACTCCTGTGCAGTTCGCCTTCGTGTTCACCGTGGGCTACATCGGCTA<br>CTTCATCTTCCGATCTGTGAACCACCAGAAGGACATCGTCCGATCTACCAACGGCGAGTGCATGATCT<br>GGGGCAAGCCCGCCAAGGTGATCCGAACCTCTTTCGTGACCTCTGACGGCAAGACCCACAAGTCTCT<br>GCTGCTGTGCTCTGGCTACTGGGGCCTGTCTCGACACTTCAACTACGTGGGCGACCTGCTCATCTCTCT<br>GGCCATGTGCATGACCTGCGGCACCCAGCATCTGCTGCCCTACTTCTACATCATCTACATGACCATCC<br>TGCTGCTCCACCGAATCCAGCGAGATCACACCCGATGCAAGGGCAAGTACGGAAAGTACTGGGACGA<br>GTACATGAAGGCCGTGCCTTACAAGCTGATCCCCTACGTGTACTAA
Glycine soja delta-7 sterol reductase	ATGGGCGCTACCGTGCACTCTCCCCTGGTGACCTACGCCTCTGTGATCTCTCTGCTGACCCTGTGTCCT<br>CCTTTCGTGGTGCTGCTGTGGTACACCATGACTCTGGCCGACGGCTCTGTGTCTGAGACTTTCCACTAC<br>CTGCGACAGAACGGCCTGCAGGGCCTGCTGCACATCTGGCCCACTCCTACTCCTACCGCCTGCAAGAT<br>CATTGCCGTGTACGCCGCCTTCGAGGCCGCTCTGCAGCTGCTGCTGCCCGGCAAGACCGTGTACGGCC<br>CCATCTCTCCCACCGGCCACCGACCTGTGTACAAGGCCAACGGACTGCAGGCCTACTTCGTGACCCTG<br>ATCACCTACTTCGCCCTGTGGTGGTTCGGCATCTTCAACCCCACCATCGTGTACCACCACCTGGGCGA<br>GATCTACTCTGCCCTGATCTTCGGCTCTTTCCTGTTCTGCGTGTTCCTGTACATCAAGGGCCATCTGGC<br>TCCCTCTTCTACCGACTCTGGATCTTCTGGCAACCTGATCATCGACTTCTACTGGGGCATGGAACTGTA<br>CCCTCGAATCGGCAAGCACTTCGACATCAAGGTGTTCACCAACTGTCGATTCGGCATGATGTCTTGGG<br>CCGTGCTGGCCCTGACCTACTGCATCAAGCAGTACGAAGAGAACGGCAAGGTGGCCGACTCTATGCT<br>GGTCAACACCGCTCTGATGCTGGTGTACGTGACCAAGTTCTTCTGGTGGGAGGCCGGCTACTGGTCTA<br>CCATGGACATTGCCCACGACCGAGCCGGCTTCTACATCTGCTGGGGCTGCCTGGTGTGGGTGCCCTCT<br>GTGTACACCTCTCCTGGCATGTACCTGGTGAACCATCCTGTGAACCTGGGCATCAAGCTGGCTCTGTC<br>TATCCTGGTGGCCGGCATCCTGTGCATCTACATCAACTACGACTGCGACCGACAGCGACAAGAGTTCC<br>GACGAACTAACGGAAAGGGCACCGTGTGGGGCAAGGCCCCTTCTAAGATCGAGGCCACCTACACCAC<br>TACCTCTGGCGAGACTAAGCGATCCCTGCTGCTGACCTCTGGCTGGTGGGGCCTGTCTCGACACTTCC<br>ACTACGTGCCCGAGATCCTGGCCGCCTTCTTCTGGACCGTGCCTGCTCTGTTCGAGCACTTCCTGCCTT<br>ACTTCTACGTGATCTTCCTGACCATCCTGCTGTTCGACCGAGCTAAGCGAGATGACGACCGATGCCGA<br>TCTAAGTACGGCAAGTACTGGAAGCTGTACTGCGACAAGGTGCCCTACCGAATCATCCCCGGCATCT<br>ACTAA
Tetraselmis sp. GSL018 delta-7 sterol reductase	ATGAAGCGAGCCTCCAAGACCCCCGACACCGCCTCTAAGGGTCGAGAACCCCTTTCTGAGCCTCACA<br>CCAACGGTGTCGCTAAGGCCAGCAACAAGACCTCTTGGGCCGAGTCCAATGGCATCGGTGATCGAGA<br>CGGATTCATGGGCCTGTCCGGTGCCGCCGGCCATGCTGTGGCCCTTCTGGGCACCGTCGTGCTGCTCG<br>TCGGTTGCCCCGCCTTTGTCTTCGTGCTCTGGTACATTAATTGTCGACTCGACGGCTCCGTCTCCGAGT<br>TCGTCGCCCTCGCCGCTCGAGAGGGCGCCGTGGGTCTCTGGCAACGATGGCCTACTCCCACTGCTGAG<br>GCCTGGGCCATCATTGGCACCTTCGGTGCAGTCGAGGCCTTCCTGCAGCTGGCTCTCCCTGGCAAGAA<br>GTTCCTGGGTCCCGTCTCTCCTAAGGGTAACGTCCCCGTCTACAAGGCCAACGGCATGCAAGCCTACG<br>TGACTACCCTGGTCCTGTTCTTTGCCGTCTGGGGCTCCGGCATCTACAACCCCGCGCGAGTCTACGAT<br>CTCATGGGTGAGATTCTGGCCGCCCTGAACATCTTTTCTCTGCTGTTTTGCCTGTTCCTCAACATTAAG<br>GGTCATGTCGCCCCTTCCTCTACTGACTCCGGCTCTACCGGCTCTTTGCTGTACGACTACTACTGGGGC<br>ATGGAGCTTTACCCTAGAATTGGCCGATCCTTCGACATTAAGACCTGGACCAACTGCCGAGTCGGCAT<br>GATGGGTTGGGGCATCCTGATCCTGTGCTACGCCGCCAAGCAGGTGGAGGAGGCTGGATTTCTGTCC<br>GACTCCATGGCTGTGTCCGTCATTCTCATGCACGTTTACATCGCCAAGTTCTTCTGGTGGGAGACTGG<br>TTACTGGAAGACCATGGACATCATGCACGATCGAGCCGGTTACTACATCTGCTGGGGTTGCCTCGTGT<br>GGATCCCCTCCATGTACACCTCTCCCACCATGTTTCTTGTCAAGCATCCTATGGTGCTGGGCCCCACCC<br>TCACCGGCGCTGTCCTGGCTGCTGGCCTGCTGTGTATCTACATCAACTACGACGCCGACCGACAGCGA<br>CAGGTTTTCCGAGAGTCTAACGGCAAGGCCCTGATTTGGGGTCGAAAGCCTAAGAAGATCGAGGCCC<br>AGTACACTACCGCGGACGGCCAAACTAAGACCTCTCTGCTGCTGGTGTCTGGTTGGTGGGGTGTCTCC<br>CGACATTTCCACTACCTGCCCGAGATACTTGCCTCCGTGTTCTGGTCTGTGCCCGCCCAGACTGATTAC<br>GCTATGGCCTACCTGTACTCTGCCTACCTCACCATTCTTCTCGTGGACCGTGCCTTCCGAGACGACCTG

	CGATGCGCTTCCAAGTACGGCAAGCACTGGGTCGAGTATTGCCGACAGGTCCCCCACAAGATCGTGC<br>CCTACATCTTCTAA
Waddlia chondrophila delta-7 sterol reductase	ATGGCCGCCACCACCACCAACGTGCAGACCCGAAACTGGGGCCGAGCCTGGGAGACTACCTGGCTGT<br>CTCTGTTCTCTACCATTGCTCTGCTGGCCACCGCTCCTATGATGGTGCTGTACTGCTACATTGCCTGCG<br>TGCGATTCCGAGGCTCTCTGATCGGACCCGCCTACGCTCTGGCCTCTGGCGCCGTGTCTCTGGACTCT<br>CTGTTCCCCTCGTTCGAGGTGGGCATCTTCGCCCTGTACCTCGGCTGGTTCGCCTTCCAGCTGCTGCTG<br>TACCTGGGACTGCCCGACCTGCTGCACCGAATTCTGCCCCGATACCGAGGCGGCCGACAAGAGGGCG<br>CTGTGACCCCTGCCGGCAAGCAGCTGGTGTACCAGATCAACGGACTGCAGGCCTGGCTGATCTCCCA<br>CCTGTTCTTCGGCATCGGCGCCTACGTGCTCGGATGGTTCTCTCCCTCGATCATTGCCGAGAACTGGG<br>GAGGCTTCCTGATCGTGACCAACGTGATGGGCTACCTGACCGCCATCTTCGTGTACGTGAAGGCCTAC<br>CGATTTCCTTCGAACGCCGAGGACCGAAAGTTCTCTGGCAACCCTCTGTACGACTTCTTCATGGGCAT<br>CGAGTTCAACCCTCGAATCGGCAAGTTCGACTTCAAGCTGTTCTTCAACGGACGACCCGGCATCATTG<br>CTTGGACCCTGATCAACTGGTCTTTCGCCGCCAAGCAGTACGCCGACCTGGGATACCTGCCTAACTCT<br>ATGCTGCTGGTGAACGTGCTGCAGGCTATCTACGTGCTGGACTTCTTCTGGCACGAGACTTGGTATCT<br>CAAGACCATCGACATCTGCCACGACCACTTCGGCTGGATGCTGTCTTGGGGCGACCTGGTGTGGCTGC<br>CCTACATGTACACCCTCCAGGGCCTGTACCTGCTCTACCATCCTGTGGACCTGTCTACCGGCTTCGCTC<br>TGTTCGTGCTGACCCTGGGCGTCGTGGGCTACGCCATTTTCCGATCTGCCAACCACCAGAAGGACCAC<br>TTCCGACGAGTGCAAGGCAAGGAACCCATCTGGGGAAAGATGCCCGAGTTCATCTCTTGCCAGTATA<br>CCGCCGCTGACGGCTCTCTGCACCACACCAAGCTGCTCCTCTCCGGCTGGTGGGGACGAGCCCGACA<br>CATGAACTACACCGGCGACCTGATGCTGTCCCTGGCCTACTGCCTGGCCTGCGGCTTCTCTCACCTCC<br>TGCCTTACTTCTACTTCGTCTACATGACCATCCTGCTGGTCAACCGATGCTACCGAGATGAGCACCGA<br>TGCGAGAACAAGTACGGCGACGCCTGGCGAAAGTACTGCCGACGAGTCCCCTACCGACTGATCCCCG<br>GCATCTACTAA
Tetrahymena thermophilia squalene-tetrahymanol cyclase	ATGAAGAAGATCCTCATCGGTCTCATCATCGGTCTCTTCCTCTTCTCCTCCGTCAACGCCTCCGTCAAC<br>CTCACCGAGGTCCAGAACGCCATCTCCATCCAGCAGGGTATCAACTGGGCTGAGGTCCACAACAACA<br>CCTGGTACTACCCTCCCTACCTCGGTGAGATGTTCATCTCCGAGTACTACTTCGAGCTCCTCGTCCTCA<br>ACTGGACCCACAAGTCCGCCTTCAACGCCACCTACTTCACCGAGCGACTCCTCCAGACCCAGTTCGAG<br>GACGGTTCCTGGGAGCAGGTCCGAGAGCAGAACCTCGAGACCGGTCAGCTCGACGCCACCGTCTTCA<br>ACTACTGGTACCTCAAGTCCATCAACAACAACCCCAAGATCGAGGCTGCCCTCCAGAAGGCCCGAAA<br>GTGGATCGTCGCTCAGGGTGGTATCGAGGCCACCCAGACCATGACCAAGTTCAAGCTCGCTGCCTTC<br>GGTCAGTACTCCTGGGAGGACCTCTGGTACGTCCCTCTCTTCATCTTCAAGCAGAACGGTATCTTCAA<br>GTACACCTACGTCAAGGACATCGTCGCCCAGTGGGTCTACCCCCACCTCACCGCTCTCGCCTACCTCC<br>GATACCAGCGAACCGTCTTCAACGTCCCTGTCGCTGACCTCCGAGAGCTCTGGATCAACTACCCCAAG<br>AACGGTATCAAGATCTCCCCCCGAGAGTACTCCACCCTCAACCCCGACTCCGACCTCCTCATCCTCAT<br>GGACGAGATCTTCAAGCTCAAGCAGCCTCTCGGTTCCTTCGGTGCCTACACCATCTCCACCCTCCTCA<br>CCCTCATGTCCTTCAAGGACTTCCAGTCCAAGCACCCCCACCTCTACCAGAACGAGATCCAGAAGGCC<br>TACGAGGACGGTTACTACTTCGTCGAGTTCAACTACTTCAACTTCCGAGAGGCCTACCACGGTTCCCT<br>CGACGACGGTCGATGGTGGGACACCATCCTCATCTCCTGGGCCATGCTCGAGTCCGGTCAGGACAAG<br>GAGCGAATCTTCCCCATCGTCCAGAACATGGTCAAGGAGGGTCTCCAGCCCAAGAAGGGTATCGGTT<br>ACGGTTACGACTTCGAGTACGCTCCCGACACCGACGACACCGGTCTCCTTCTCGTCGTCATGTCCTAC<br>TACAAGGAGGCCTTCCAGAAGCAGATCCCCGAGACCATCGAGTGGCTCTTCTCCATGCAGAACGACG<br>ACGGTGGTTACCCCGCCTTCGACAAGGGTAAGAACGAGGACAACCTCCTCTTCAAGTTCGCCTTCAAC<br>ATGGCCGGTATCGCCAACTCCGCCGAGATCTTCGACCCTTCCTGCCCTGACATCACCGGTCACATCAT<br>GGAGGGTCTCGGTGAGTTCGGTTACCAGGCCAACCACCCCCAGATCCAGAACATGATCAAGTACCAG<br>CGAAAGACCCAGAACAAGTGGGGTTCCTGGCAGGCTCGATGGGGTGTCAACTACATCATGGCTGTCG<br>GTGCTGTCGTTCCTGGTCTCGCTCGAGTCAACTACGACCTCAACGAGCAGTGGGTCCAGAACTCCATC<br>AACTACCTCCTCAACAAGCAGAACAAGGATGGCGGCTTCGGTGAGTGCGTCCTCTCCTACAACGACC<br>CCGAGAAGTGGAACGGTATCGGTAAGTCCACCGTCACCCAGACCTCCTGGGGTCTCCTCGCTCTCCTC<br>GAGGTCTACAACCAGAACGAGCAGATCAAGCACGCTGCCGATCGAGCTGCCCAGTACCTCCTCGACC<br>AGTTCAAGCGAGACGACAACACCTTCTACGACCACTCCACCATCGGTACCGGTCACCGAGGTCTCCTC<br>TACCTCCAGTACCCCTCCTACGCCCAGTCCTTCCCTCTCGTCGCCCTCAACCGATACCAGAAGATCTCC<br>CAGGGTCAGTACCACTTCTCCAAGAACCTCTACAACGGTAACGGTGAGCCCGTCCAGAAGCAGAACA<br>TCTAA
Solanum lycopersicum delta-24(25) sterol reductase	ATGTCTGACGCCAAGGCTCCCGTGGCCACTGCTTACCCCAAGCGAAAGATCCAGCTGGTGGACTTCCT<br>GCTGTCTTTCCGATGGATCATCGTGATTTTCTTCGTGCTGCCCTTCTCTTTCCTGTACTACTTCTCTATC<br>TACCTGGGCGACGTGAAGTCTGAGCGAAAGTCTTACAAGCAGCGACAGATGGAACACGACGAGAAC<br>GTGAAGGAAGTGGTGAAGCGACTGGGCCAGCGAAACGCCGAGAAGGACGGCCTGGTGTGCACCGCT<br>CGACCTCCATGGGTCGTCGTGGGCATGCGAAACGTGGACTACAAGCGAGCCCGACACTTCGAGGTGG<br>ACCTGTCTAAGTTCCGAAACATCCTGGACATCGACACCGAGCGAATGGTGGCCAAGGTCGAGCCCCT<br>GGTGAACATGGGCCAGATGTCTCGAGTGACCATTCCTATGAACCTGTCTCTGGCCGTGCTGGCCGAGC<br>TGGACGACCTGACCGTCGGCGGCCTGATCAACGGCTTCGGCGTCGAGGGATCTTCTCACATCTTCGGC<br>CTGTTCTCTGACACCGTGGTGGCCCTCGAGGTGGTGCTGGCTGACGGCAAGGTGGTGCGAGCCACCA<br>AGGACAACGAGTACTCTGACCTGTTCTACGCTATCCCCTGGTCGCAGGGCACCCTGGGCCTGCTGGTG<br>TCTGCCGAGATCAAGCTGATCCCCGTGGACCAGTACGTGAAGCTGACCTACAAGCCCGTGCGAGGCA<br>ACCTGAAGGAACTGGCCCAGGCCTACGCCGACTCTTTCGCTCCCAAGGACGGCGACCAGGACAACCC

	CTCTAAGGTGCCCGAGATGGTCGAGGGCATGATCTACGGCCCCACCGAGGGCGTGATGATGACCGGC<br>ATGTACGCCTCTCGAAACGAGGCCAAGCGACGAGGCAACGTGATCAACAACTACGGCTGGTGGTTCA<br>AGCCCTGGTTCTACCAGCACGCTCAGACCGCTCTGAAGCGAGGCGAGTTCGTCGAGTACATCCCTACT<br>CGAGACTACTACCACCGACACACCCGATCTCTGTACTGGGAGGGCAAGCTGATTCTGCCCTTCGGTGA<br>CCAGTTCTGGTTCCGATTCCTGCTCGGCTGGCTGATGCCTCCTAAGATCGCCCTGCTGAAGGCTACCC<br>AGTCTGAGGCCATCCGAAACTACTATCACGACCACCACGTGATCCAGGACCTGCTCGTGCCCCTGTAC<br>AAGGTGGGCGACTGCCTCGAGTGGGTGCACCGAGAGATGGAAGTGTACCCCATCTGGCTGTGTCCCC<br>ACCGAATCTACAAGCTGCCTGTGCGACCTATGATCTACCCCGAGCCTGGCTTCGAGAAGCACAAGCG<br>ACAGGGTGACACCGAGTACGCCCAGATGTACACCGACGTGGGCGTGTACTACGTGCCCGGTGCCGTG<br>CTGCGAGGTGAGCCCTTCGACGGCTCTGAGAAGTGCCGACAGCTGGAACTGTGGCTGATCGAGAACC<br>ACGGCTTCCAGGCTCAGTACGCCGTGACCGAGCTGACCGAGAAGAACTTCTGGCGAATGTTCGACAA<br>CGGCCTGTACGAGCAGTGCCGACGAAAGTACAAGGCCATCGGCACCTTCATGTCTGTGTACTACAAG<br>TCTAAGAAGGGCCGAAAGACTGAGAAGGAAGTTCAAGAGGCCGAGCAAGAGAAGGCTGAGCAAGA<br>AACCCCTGAGGCCAACTAA
Chenopodium quinoa sterol methyl transferase	ATGGACTCTATGGCCCTGATCTGCACCGTGGGCCTGCTGTTCGGCGGCCTGTACTGGTTCATCTGCAT<br>TCACGGACCCGCCGAGCGAAAGGGCAAGCGAGCCGTGGACCTGTCTGGCGGCTCTATCTCTTCTGAC<br>AAGGTGCAGGACAAGTACCAGCAGTACTGGTCGTTCTTCCGACGACCTAAGGAAATCGAGACTGCCG<br>AGAAGGTGCCCGACTTCGTGGACACCTTCTACAACCTGGTGACCGACATCTACGAGTGGGGCTGGGG<br>CCAGTCTTTCCACTTCTCTCCCTCGATTCCCGGCAAGTCTCACCGAGATGCTACCCGAATCCACGAAG<br>AGATGGCCGTCGACCTGATCAAGGTGTCTCCCGGCCAAAAGATCCTGGACGTCGGCTGCGGCGTCGG<br>CGGACCCATGCGAGCCATTGCCGCTCACTCTCGAGCCAAGGTGACCGGCATCACCATCAACGAGTAC<br>CAGGTGAAGCGAGCCAAGCTGCACAACAAGAAGGCCGGACTGGACTCTCTGTGCGAGGTGGTGTGC<br>GGCAACTTCCTCGAGATGCCCTTCGCCTCTAACACCTTCGACGGCGCCTACTCTATCGAGGCCACCTG<br>TCACGCTCCCAAGCTGGACGACGTGTACTCTGAGATCTTCCGAGTGCTGAAGCCCGGCTCTCTGTACG<br>TGTCTTACGAATGGGTGACCACCGACAAGTTCAACGGCGACGACTCTGAGCACTGCGACGTGATCCA<br>GGGCATCGAGCGAGGCGACGCTCTGCCCGGCCTGCGACGATACGACGAGATCTCTGAGGCCGCCAAG<br>AAGGTGGGCTTCGAGATCGTGGACGAGCGAGATCTGGCCGCTCCTCCTGCTAAGCCCTGGTGGGACC<br>GACTGAAGATGGGCCGAATCGCCTACTGGCGAAACCACATCGTGGTGACCGTGCTGGCCGCCATCGG<br>CGTGGCTCCCAAGGGCACCGTGGACGTGCACGACATGCTGTTCAAGACCGCCGACTACCTGACTCGA<br>GGCGGCGAGTCTGGCATTTTCTCTCCCATGCACATGATCCTGTGTCGAAAGCCCGTGGACGCCAAGTC<br>TGACTCTTAA
Solanum tuberosum delta-24(28) sterol reductase	ATGACCGACGTCCAGGCCCCTCCTCGACCCAAGCGAAAGAAGAACATCATGGACCTCCTCGTCCAGT<br>TCCGATGGATCGTCGTCATCTTCGTCGTCCTCCCCCTCTCCTTCCTCTACTACTTCTCCATCTACGTCGG<br>TGACGTCCGATCCGAGTGCAAGTCCTACAAGCAGCGACAGAAGGAGCACGACGAGAACGTCAAGAA<br>GGTCGTCAAGCGACTCAAGGACCGAAACGCCTCCAAGGACGGTCTGGTCTGCACCGCCCGAAAGCCC<br>TGGGTCGCTGTCGGTATGCGAAACGTCGACTACAAGCGAGCCCGACACTTCGAGGTCGACCTCTCCC<br>CCTTCCGAAACGTCCTCAACATCGACACCGAGCGAATGATCGCCAAGGTCGAGCCCCTCGTCAACAT<br>GGGTCAGATCTCCCGAGTCACCGTCCCCATGAACGTCTCCCTCGCCGTCGTCGCTGAGCTCGACGACC<br>TCACCGTCGGTGGTCTGATCAACGGTTACGGTATCGAGGGTTCCTCCCACATCTACGGTCTGTTCTCC<br>GACACCGTCGTCTCCTACGAGGTCGTCCTCGCCGACGGTCAGGTCGTCCGAGCCACCAAGGACAACG<br>AGTACTCCGACCTCTTCTACGCCATCCCCTGGTCCCAGGGTACCCTCGGTCTGCTCGTCTCCGCCGAG<br>ATCAAGCTCATCCCCATCAAGGAGTACATGAAGCTCACCTACAAGCCCGTCGTCGGTAACCTCAAGG<br>AGATCGCCCAGGCCTACATCGACTCCTTCTCCCCCAAGGACGGTGACCAGGACAACCGAGAGAAGGT<br>CCCCGACTTCGTCGAGACTATGGTCTACACCCCCACCGAGGCTGTCTGCATGACCGGTCGATACGCCT<br>CCAAGGAGGAGGCCAAGAAGAAGGGTAACGTCATCAACAACGTCGGTTGGTGGTTCAAGACCTGGTT<br>CTACCAGCACGCCCAGACCGCTCTCAAGAAGGGTGAGTTCGTCGAGTACATCCCCACCCGAGAGTAC<br>TACCACCGACACACCCGATGCCTCTACTGGGAGGGTAAGCTCATCCTCCCCTTCGGTGACCAGTGGTG<br>GTTCCGATTCTTCTTCGGTTGGGCCATGCCCCCTAAGGTTTCCCTCCTCAAGGCCACCCAGGGTGAGT<br>ACATCCGAAACTACTACCACGAGAACCACGTCATCCAGGACATGCTCGTCCCCCTCTACAAGGTCGG<br>TGACGCCCTCGAGTGGGTCAACCGAGAGATGGAGGTCTACCCCCTCTGGCTCTGCCCCCACCGACTCT<br>ACCGACTCCCCCTCAAGACCATGGTCTACCCCGAGCCTGGTTTCGAGCTCCACAAGCGACAGGGTGA<br>CACCAAGTACGCCCAGATGTACACCGACGTCGGTGTCTACTACGCCCCCGGTCCCATCCTCCGAGGTG<br>AGGTCTTCGACGGTATCGAGGCCGTCCGAAAGCTCGAGTCCTGGCTCATCGAGAACCACGGTTTCCA<br>GCCCCAGTACGCCGTCTCCGAGCTCACCGAGAAGAACTTCTGGCGAATGTTCGACGGTTCCCTCTACG<br>AGAACTGCCGAAAGAAGTACCGAGCCATCGGTACCTTCATGTCCGTCTACTACAAGTCCAAGAAGGG<br>TAAGAAGACCGAGAAGGAGGTCCAGGACGCCGAGCAGGAGACTGCCGAGGTCGAGACTCCCGAGGT<br>CGACGAGCCCGAGGACTAA

Supplementary Table 4.
Plasmids used for strain engineering in this study. The corresponding parental plasmids and BioBricks that were used to construct each plasmid are given.

**Plasmid name**	**Parent plasmid/ Reference**	**BioBricks**
pCfB6611 (pNat-PrExp-Cre)	pCfB4158 (pPrExp-Cre), Holkenbrink *et al,* 2018 [31]	pCfB4158 backbone: BB1037(Fragment1EpiVecYL)
pCfB6633 (pNat-YLgRNA2_IntE_1)	Holkenbrink *et al,* 2018[31]	
pCfB6637 (pNat-YLgRNA3_IntE_3)	Holkenbrink *et al,* 2018[31]	
pCfB6638 (pNat-YLgRNA2_IntE_4)	Holkenbrink *et al,* 2018[31]	
pCfB6677 (pIntE_1-TPex20-TLip2)	Holkenbrink *et al,* 2018[31]	
pCfB6679 (pIntE_4-TPex20-TLip2)	Holkenbrink *et al,* 2018[31]	
pCfB6681 (pIntE_3-TPex20-TLip2)	Holkenbrink *et al,* 2018[31]	
pBP8003 (pNat-YLgRNA4-IntF_3)	Dr. K.R. Kildegaard, Biophero ApS, Denmark	
pBP8009 (pIntF_3-TPex20-TLip2)	Dr. K.R. Kildegaard, Biophero ApS, Denmark	
pBP8660 (pIntE_5-TPex20-TLip2)	Dr. K.R. Kildegaard, Biophero ApS, Denmark	
pCfB8861 (pHyg-YLgRNA3_IntE_4)	Arnesen *et al,* 2020[23]	
pCfB10249 (IntE4_PrTEFInt->DHCR7_Stuberosum)	pCfB6679	BB3879 (TefInt->):BB4876 (PrTEFInt_DHCR7St)
pCfB10250 (IntE4_PrTEFInt->DHCR7_Drerio)	pCfB6679	BB3879 (TefInt->):BB4877 (PrTEFInt_DHCR7Dr)
pCfB10251 (IntE4_PrTEFInt->DHCR7_Ldrancou)	pCfB6679	BB3879 (TefInt->):BB4878 (PrTEFInt_DHCR7Ld)
pCfB10252 (IntE4_PrTEFInt->DHCR7_Esilicul)	pCfB6679	BB3879 (TefInt->):BB4879 (PrTEFInt_DHCR7Es)
pCfB10253 (IntE4_PrTEFInt->DHCR7_Cprotoch)	pCfB6679	BB3879 (TefInt->):BB4880 (PrTEFInt_DHCR7Cp)
pCfB10254 (IntE4_PrTEFInt->DHCR7_Csubellip)	pCfB6679	BB3879 (TefInt->):BB4881 (PrTEFInt_DHCR7Cs)
pCfB10255 (IntE4_PrTEFInt->DHCR7_Mverticillata)	pCfB6679	BB3879 (TefInt->):BB4882 (PrTEFInt_DHCR7Mv)
pCfB10256 (IntE4_PrTEFInt->DHCR7_Gsoja)	pCfB6679	BB3879 (TefInt->):BB4883 (PrTEFInt_DHCR7Gs)
pCfB10257 (IntE4_PrTEFInt->DHCR7_Tsp)	pCfB6679	BB3879 (TefInt->):BB4884 (PrTEFInt_DHCR7Ts)
pCfB10258 (IntE4_PrTEFInt->DHCR7_Wchondrophi)	pCfB6679	BB3879 (TefInt->):BB4885 (PrTEFInt_DHCR7Wc)
pCfB10494 (IntE3_PrGPAT->TtSTC)	pCfB6681	BB1617 (PrGPAT): BB5100 (TtSTC_USER)
pCfB10783(gRNA_IntE_5)	Dr. J. Dahlin, DTU CfB, Denmark	
pCfB10851 (IntE1_PrGPAT->SMT2_Cquinoa)	pCfB6677	BB1617 (PrGPAT->): BB5371 (PrGPAT_SMT2Cq)
pCfB10853 (IntE5_PrGPAT->DHCR24_Slycopersic)	pBP8660	BB1617 (PrGPAT->): BB5373 (PrGPAT_DHCR24Sl)
pCfB10948 (IntF3_PrDGA1->StSSR1)	pCfB8009	BB1616 (PrDGA1): BB5456 (StSSR1_USER)

Supplementary Table 5.

BioBricks used in this study. The DNA template and primers used for the amplification of each BioBrick are given.

**BioBrick name**	**Template/Reference**	**Forward primer**	**Reverse primer**
pCfB4158 backbone	pCfB4158, Holkenbrink *et al,* 2018[31]	10593	10594
BB1037(Fragment1EpiVecYL)	Holkenbrink *et al,* 2018[31]	10591	10592
BB1616 (PrDGA1->)	Holkenbrink *et al,* 2018[31]		
BB1617 (PrGPAT->)	Holkenbrink *et al,* 2018[31]		
BB3879 (PrTefInt->)	Arnesen *et al,* 2020[23]		
BB4876 (PrTEF_DHCR7St)	*Solanum tuberosum* delta-7 sterol reductase codon optimised	PR-27565	PR-27566
BB4877 (PrTEF_DHCR7Dr)	*Danio rerio* delta-7 sterol reductase codon optimised	PR-27567	PR-27568
BB4878 (PrTEF_DHCR7Ld)	*Legionella drancourtii* delta-7 sterol reductase codon optimised	PR-27569	PR-27560
BB4879 (PrTEF_DHCR7Es)	*Ectocarpus siliculosus* delta-7 sterol reductase codon optimised	PR-27571	PR-27572
BB4880 (PrTEF_DHCR7Cp)	Candidatus *Protochlamydia amoebophila* delta-7 sterol reductase codon optimised	PR-27573	PR-27574
BB4881 (PrTEF_DHCR7Cs)	*Coccomyxa subellipsoidea* delta-7 sterol reductase codon optimised	PR-27575	PR-27576
BB4882 (PrTEF_DHCR7Mv)	*Mortierella verticillate* delta-7 sterol reductase codon optimised	PR-27577	PR-27578
BB4883 (PrTEF_DHCR7Gs)	*Glycine soja* delta-7 sterol reductase codon optimised	PR-27579	PR-27570
BB4884 (PrTEF_DHCR7Ts)	*Tetraselmis* sp. GSL018 delta-7 sterol reductase codon optimised	PR-27581	PR-27582
BB4885 (PrTEF_DHCR7Wc)	*Waddlia chondrophila* delta-7 sterol reductase codon optimised	PR-27583	PR-27584
BB5041 (LoxP_HphMX)	pCfB5935, Holkenbrink *et al,* 2018[31]	PR-27972	PR-27973
BB5091 (*ERG4*_1kbUP_U1)	ST9100 gDNA	PR-27891	PR-28358
BB5092 (*ERG4*_1kbDOWN_U2)	ST9100 gDNA	PR-27893	PR-27894
BB5093 (*ERG5*_1kbUP_U1)	ST9100 gDNA	PR-27895	PR-28359
BB5094 (*ERG5*_1kbDOWN_U2)	ST9100 gDNA	PR-27897	PR-27898
BB5097 (*ERG4*_HphMX_KO)	BB5091 + BB5092 + BB5041 USER reaction	PR-27891	PR-27894
BB5098 (*ERG5*_HphMX_KO)	BB5093 + BB5094 + BB5041 USER reaction	PR-27895	PR-27898
BB5100 (TtSTC_USER)	*Tetrahymena thermophilia* squalene-tetrahymanol cyclase codon optimised	PR-28361	PR-28362
BB5371 (PrGPAT_SMT2Cq)	*Chenopodium quinoa* sterol methyl transferase codon optimised	PR-27621	PR-27786
BB5373 (PrGPAT_DHCR24Sl)	*Solanum lycopersicum* delta-24(25) sterol reductase codon optimised	PR-27599	PR-27764
BB5456 (StSSR1_USER)	*Solanum tuberosum* delta-24(28) sterol reductase codon optimised	PR-29404	PR-29405

Supplementary Table 6.
Primers used in this study.

Primer name	Sequence (5' → 3')
PR-14617 (vector verification *E. coli* cPCR)	tatccctgtgttgaatc
PR-14619 (vector verification *E. coli* cPCR)	tatcgacccagttagc
PR-8859 (integration verification Yl cPCR)	aagtgtggatggggaagtgag
PR-14442 (IntE_1 verification Yl cPCR)	agttgtgaccaagacaaatg
PR-14398 (IntE_1 verification Yl cPCR)	cacgcgaUgttagaagcaattggagaag
PR-14576 (IntE_3 verification Yl cPCR)	cacgcgautgaaggaaatgcctaaaacc
PR-14835 (IntE_3 verification Yl cPCR)	cacgcacgccattctataag
PR-14592 (IntE_4 verification Yl cPCR)	acgcgauttaacactggaccgtactgc
PR-20880 (IntE_4 verification Yl cPCR)	attgctaagcgaccatagac
PR-14837 (IntF_3 verification Yl cPCR)	acatgctcgcgcctcgatag
PR-14584 (IntF_3 verificiationYl cPCR)	cacgcgautttggtcgtcgcccaacaag
PR-26834 (IntE_5 verification Yl cPCR)	gggacaagtgcaatcgttcacttg
PR-26835 (IntE_5 verification Yl cPCR)	cgttgctgttgccacaatggac
PR-27891 (Erg4Rep_P1F)	ctctcaacaccttcaccgc
PR-28358 (Erg4Rep_P1R_U1)	atcgcacgugataagcttagtgagcgaatgg
PR-27893 (Erg4Rep_P2F)	gtgcaggutgtgtggttgcgaaggaag
PR-27894 (Erg4Rep_P2R)	gcactcaaaataccccgttc
PR-27895 (Erg5Rep_P1F)	ctcggtttgttgcagcagg
PR-28359 (Erg5Rep_P1R_U1)	atcgcacgutggtccgtatcgtgaaatgg
PR-27897 (Erg5Rep_P2F)	gtgcaggugggcggagttgtgttgtg
PR-27898 (Erg5Rep_P2R)	ggtcggctatccaatacatctc
PR-27972 (HphMX_F_U1)	cgtgcgautcagctgaagcttcgtac
PR-27973 (HphMX_R_U2)	acctgcacugcataggccactagtgg
PR-28361 (TtSTC_F_U3)	atctgtcaugccacaatgaagaagatcctcatcggtc
PR-28362 (TtSTC_Rv)	cacgcgauttagatgttctgcttctggacg
PR-22830 (erg5Δ_chk)	tcatactcaccgaaacgtg
PR-27961 (erg5Δ_chk)	gttccaatgcctggcaag
PR-27634 (erg5Δ_chk)	acttctctctctcacaccacc
PR-27635 (erg5Δ_chk)	ctgagggctctgttggtgaag
PR-27636 (erg5Δ_chk)	accagtgtggttgtaaggatg
PR-11138 (erg4Δ_chk)	agcaatggguaaaaagcctgaactcaccgc
PR-27631 (erg4Δ_chk)	cctgatattggtgatcctcc
PR-27632 (erg4Δ_chk)	agagccttgtttccgaggtg
PR-27633 (erg4Δ_chk)	atacaatcccataggctggc
PR-28001 (erg4Δ_chk)	cgtgcgaugcttgccctggactacatcttg
PR-27565 (A_St_Fw)	actttttgcagtacuaaccgcaggccgagtctcagctggtgcac
PR-27566 (A_St_Rv)	cacgcgauttagtagatgccggggatcac
PR-27567 (A_Dr_Fw)	actttttgcagtacuaaccgcagatggcctctgaccgagttcg
PR-27568 (A_Dr_Rv)	cacgcgauttagaagatgttgggcagcag
PR-27569 (A_Ld_Fw)	actttttgcagtacuaaccgcagtacttcaagatccgaaacac
PR-27570 (A_Ld_Rv)	cacgcgauttagatcacgaagggcacgatc
PR-27571 (A_Es_Fw)	actttttgcagtacuaaccgcagatcgacggcgctgccatcg
PR-27572 (A_Es_Rv)	cacgcgauttacaggatgccgggcacgatc
PR-27573 (A_Cp_Fw)	actttttgcagtacuaaccgcagctgatcgagatgctgtgcatc
PR-27574 (A_Cp_Rv)	cacgcgauttagatcacgaaggggatgatc
PR-27575 (A_Cs_Fw)	actttttgcagtacuaaccgcaggtcacaacccgagccgctg
PR-27576 (A_Cs_Rv)	cacgcgauttagaaaatgtaagggatcatc
PR-27577 (A_Mv_Fw)	actttttgcagtacuaaccgcaggccgtgcagcagcgaaagac
PR-27578 (A_Mv_Rv)	cacgcgauttagtacacgtaggggatcagc
PR-27579 (A_Gs_Fw)	actttttgcagtacuaaccgcagggcgctaccgtgcactctc
PR-27580 (A_Gs_Rv)	cacgcgauttagtagatgccggggatg
PR-27581 (A_Ts_Fw)	actttttgcagtacuaaccgcagaagcgagcctccaagaccc
PR-27582 (A_Ts_Rv)	cacgcgauttagaagatgtagggcacgatc
PR-27583 (A_Wc_Fw)	actttttgcagtacuaaccgcaggccgccaccaccaccaacg
PR-27584 (A_Wc_Rv)	cacgcgauttagtagatgccggggatcag

PR-27599 (B_Sl_Fw)	atctgtcaugccacaatgtctgacgccaaggctc
PR-27764 (B_Sl_Rv)	cacgcgauttagttggcctcagggg
PR-27621 (C_Cq_Fw)	atctgtcaugccacaatggactctatggccctg
PR-27786 (C_Cq_Rv)	cacgcgauttaagagtcagacttggc
PR-29404 (StSSR1_F_U3)	atctgtcaugccacaatgaccgacgtccagg
PR-29405 (StSSR1_Rv)	cacgcgauttagtcctcgggctcgtc

Supplementary Table 7.

Sterol composition of diets prepared for feeding trials (values given to 3 d.p. ± standard deviation).

	Dietary sterol content (w/w)				Percentage of total sterol (%)			
**Sterol**	**MxSt**	**WT**	**Tet**	**Base**	**MxSt**	**WT**	**Tet**	**Base**
24-Methylenecholesterol	0.146 ± 0.040	0	0	0	43.3 ± 4.15	0.300 ± 0.068	0.228 ± 0.037	0.131 ± 0.051
Campesterol	0.038 ± 0.028	0.016 ± 0.000	0.015 ± 0.002	0.069 ± 0.011	13.5 ± 10.8	14.4 ± 1.28	11.3 ± 0.647	20.1 ± 1.45
Cholesterol	0.008 ± 0.000	0	0	0.001 ± 0.000	2.61 ± 0.496	0.421 ± 0.073	0.257 ± 0.022	0.209 ± 0.015
Desmosterol	0	0	0	0	0.114 ± 0.037	0	0	0
Isofucosterol	0.014 ± 0.005	0.016 ± 0.001	0.014 ± 0.001	0.053 ± 0.011	4.6 ± 2.38	14.2 ± 0.267	10.3 ± 0.876	15.9 ± 4.28
Sitosterol	0.022 ± 0.004	0.026 ± 0.002	0.023 ± 0.002	0.073 ± 0.004	7.06 ± 2.60	23.5 ± 1.42	16.6 ± 1.54	21.6 ± 2.45
Tetrahymanol	0.073 ± 0.054	0	0.037 ± 0.006	0	19.6 ± 13.3	0	27.1 ± 3.41	0
Zymosterol	0.001 ± 0.000	0.001 ± 0.000	0	0	0.220 ± 0.175	0.574 ± 0.118	0.200 ± 0.021	0
Ergosterol	0	0.015 ± 0.003	0.013 ± 0.001	0	0	13.3 ± 2.31	9.3 ± 0.427	0
Campestanol	0.002 ± 0.002	0.007 ± 0.000	0.007 ± 0.001	0.038 ± 0.008	0.422 ± 0.597	6.26 ± 0.168	5.1 ± 0.643	11.2 ± 2.15
Cycloartenol	0.004 ± 0.000	0.003 ± 0.000	0.003 ± 0.000	0.009 ± 0.001	1.15 ± 0.308	2.56 ± 0.421	2.04 ± 0.119	2.77 ± 0.398
Stigmasterol	0.005 ± 0.001	0.004 ± 0.000	0.003 ± 0.000	0.015 ± 0.003	1.53 ± 0.119	3.24 ± 0.294	2.36 ± 0.241	4.38 ± 0.501
31-Norcycloartanol	0.005 ± 0.000	0.004 ± 0.001	0.004 ± 0.000	0.014 ± 0.002	1.47 ± 0.216	4.02 ± 0.261	3.25 ± 0.172	4.21 ± 0.484
Sitostanol	0.007 ± 0.002	0.013 ± 0.002	0.010 ± 0.001	0.044 ± 0.034	2.17 ± 0.390	11.3 ± 0.794	7.61 ± 1.33	11.8 ± 8.11
24(28)-Methylenecycloartanol	0.007 ± 0.000	0.007 ± 0.001	0.006 ± 0.001	0.026 ± 0.005	2.22 ± 0.582	5.93 ± 0.453	4.35 ± 0.127	7.69 ± 2.10
Total	0.331 ± 0.061	0.111 ± 0.007	0.136 ± 0.010	0.341 ± 0.045	100	100	100	100

References

55. Lecain, E., Chenivesse, X., Spagnoli, R. & Pompon, D. Cloning by metabolic interference in yeast and enzymatic characterization of Arabidopsis thaliana sterol Δ7-reductase. *Journal of Biological Chemistry* **271**, 10866–10873 (1996).
56. Duport, C., Spagnoli, R., Degryse, E. & Pompon, D. Self-sufficient biosynthesis of pregnenolone and progesterone in engineered yeast. *Nat Biotechnol* **16**, 186–189 (1998).
57. Souza, C. M. et al. A stable yeast strain efficiently producing cholesterol instead of ergosterol is functional for tryptophan uptake, but not weak organic acid resistance. *Metab Eng* **13**, 555–569 (2011).
58. Du, H. X. et al. Engineering Yarrowia lipolytica for campesterol overproduction. *PLoS One* **11**, e0146773 (2016).
59. Tsukagoshi, Y. et al. Ajuga Δ24-sterol reductase catalyzes the direct reductive conversion of 24-methylenecholesterol to campesterol. *Journal of Biological Chemistry* **291**, 8189–8198 (2016).
60. Zhang, Y. et al. Improved campesterol production in engineered Yarrowia lipolytica strains. *Biotechnol Lett* **39**, 1033–1039 (2017).
61. Qian, Y. D. et al. Increased campesterol synthesis by improving lipid content in engineered Yarrowia lipolytica. *Appl Microbiol Biotechnol* **104**, 7165–7175 (2020).
62. Xu, S., Chen, C. & Li, Y. Engineering of phytosterol-producing yeast platforms for functional reconstitution of downstream biosynthetic pathways. *ACS Synth Biol* **9**, 3157–3170 (2020).
63. Yang, J., Li, C. & Zhang, Y. Engineering of Saccharomyces cerevisiae for 24-Methylene-Cholesterol Production. *Biomolecules* **11**, 1710 (2021).
64. Xu, L. et al. Metabolic engineering of Saccharomyces cerevisiae for gram-scale diosgenin production. *Metab Eng* **70**, 115–128 (2022).
65. Hirz, M., Richter, G., Leitner, E., Wriessnegger, T. & Pichler, H. A novel cholesterol-producing Pichia pastoris strain is an ideal host for functional expression of human Na,K-ATPase α3β1 isoform. *Appl Microbiol Biotechnol* **97**, 9465–9478 (2013).
66. Cheng, J. et al. The origin and evolution of the diosgenin biosynthetic pathway in yam. *Plant Commun* **2**, 100079 (2021).
67. Marella, E. R. et al. A single-host fermentation process for the production of flavor lactones from non-hydroxylated fatty acids. *Metab Eng* **61**, 427–436 (2020).
