# Supplementary material for: Engineered yeast provides rare but essential pollen sterols for honeybees
Source: Nature. 2025 Aug 20;646(8084):365–71. doi: 10.1038/s41586-025-09431-y (PMC12507675; doi:10.1038/s41586-025-09431-y)
Supplement: Supplementary file 2 — Reporting Summary [file 41586_2025_9431_MOESM2_ESM.pdf]

nature portfolio

Corresponding author(s): Elynor Moore, Geraldine Wright

Last updated by author(s): Jun 22, 2025

Reporting Summary

Nature Portfolio wishes to improve the reproducibility of the work that we publish. This form provides structure for consistency and transparency in reporting. For further information on Nature Portfolio policies, see our Editorial Policies and the Editorial Policy Checklist.

Statistics

For all statistical analyses, confirm that the following items are present in the figure legend, table legend, main text, or Methods section.

n/a	Confirmed	
☐	☒	The exact sample size (n) for each experimental group/condition, given as a discrete number and unit of measurement
☐	☒	A statement on whether measurements were taken from distinct samples or whether the same sample was measured repeatedly
☐	☒	The statistical test(s) used AND whether they are one- or two-sided<br>*Only common tests should be described solely by name; describe more complex techniques in the Methods section.*
☐	☒	A description of all covariates tested
☐	☒	A description of any assumptions or corrections, such as tests of normality and adjustment for multiple comparisons
☐	☒	A full description of the statistical parameters including central tendency (e.g. means) or other basic estimates (e.g. regression coefficient) AND variation (e.g. standard deviation) or associated estimates of uncertainty (e.g. confidence intervals)
☐	☒	For null hypothesis testing, the test statistic (e.g. F, t, r) with confidence intervals, effect sizes, degrees of freedom and P value noted<br>*Give P values as exact values whenever suitable.*
☒	☐	For Bayesian analysis, information on the choice of priors and Markov chain Monte Carlo settings
☒	☐	For hierarchical and complex designs, identification of the appropriate level for tests and full reporting of outcomes
☐	☒	Estimates of effect sizes (e.g. Cohen's d, Pearson's r), indicating how they were calculated

Our web collection on statistics for biologists contains articles on many of the points above.

Software and code

Policy information about availability of computer code

Data collection	No software was used
Data analysis	R versions 4.2.2 and 4.5.0, Adobe Photoshop version 23.4.2, Chromeleon version 7.2.9, ChemStation Enhanced Data Analysis version E.01.00, ImageJ version 1.54f, GraphPad Prism 10.3.0, SPSS version 29. R packages: stats R version 4.2.2, emmeans verison 1.8.4-1, glmmTMB version 1.1.5, car version 3.1-1.

For manuscripts utilizing custom algorithms or software that are central to the research but not yet described in published literature, software must be made available to editors and reviewers. We strongly encourage code deposition in a community repository (e.g. GitHub). See the Nature Portfolio guidelines for submitting code & software for further information.

Data

Policy information about availability of data

All manuscripts must include a data availability statement. This statement should provide the following information, where applicable:

- Accession codes, unique identifiers, or web links for publicly available datasets
- A description of any restrictions on data availability
- For clinical datasets or third party data, please ensure that the statement adheres to our policy

All data is available at https://doi.org/10.6084/m9.figshare.22820849.

Research involving human participants, their data, or biological material

Policy information about studies with human participants or human data. See also policy information about sex, gender (identity/presentation), and sexual orientation and race, ethnicity and racism.

Reporting on sex and gender	N/A
Reporting on race, ethnicity, or other socially relevant groupings	N/A
Population characteristics	N/A
Recruitment	N/A
Ethics oversight	N/A

Note that full information on the approval of the study protocol must also be provided in the manuscript.

Field-specific reporting

Please select the one below that is the best fit for your research. If you are not sure, read the appropriate sections before making your selection.

☒ Life sciences ☐ Behavioural & social sciences ☐ Ecological, evolutionary & environmental sciences

For a reference copy of the document with all sections, see nature.com/documents/nr-reporting-summary-flat.pdf

Life sciences study design

All studies must disclose on these points even when the disclosure is negative.

Sample size	For honeybee feeding trials, sample sizes were informed by a power analysis previously conducted for an equivalent experimental set-up, whereby five colonies per treatment group was determined to be sufficient to observe treatment effects with 80% power (Gonçalves et al. 2025, in preparation). For the yeast feeding trial, six colonies were used per treatment group. For the pollen starvation trial, either seven or eight colonies were used per treatment group. No sample-size calculation was performed for all other experiments. For honeybee pupal sterol analysis from naturally-fed colonies, three pupae were collected for each sample to ensure sterol concentrations were detectable by GC-MS, and five samples were collected per group to account for possible variability in pupal size. For honeybee pupae and nurse sample collection during yeast feeding trials, six individuals were pooled per sample to account for possible variation in size of the individuals whilst limiting disturbance to the population dynamics of the colonies, and samples were collected from three hives. For fermentation, only two bioreactors were available at any one time, so each strain was cultivated in duplicate. Sterol analysis of yeast strains was performed in triplicate, to account for possible variation in growth between wells as well as sterol production between biological replicates.
Data exclusions	For bee feeding trials, the first month of brood counts were excluded from the main analysis as this coincided with an extreme heatwave that required us to top-up the hives with bees. Data collected after we stopped adding new bees were used for analysis.
Replication	Apart from bioreactor cultivations, a minimum of three replicates were collected for all experiments. For bioreactor cultivations, two biological replicates with two technical replicates each were performed for measurement of all parameters. Replicates gave similar results for all experiments.
Randomization	Hives used in feeding trials were randomly assigned to each feeding group. Honeybees of the appropriate types were randomly selected within hives for sterol anaylsis.
Blinding	During hive assessment, investigators were blinded to feeding group assignment. For all GC-MS and HPLC analysis, samples were assigned non-descriptive number tags, such that the origin of samples was not obvious during data measurements. For data plotting and statistical analysis, blinding measures were removed.

Reporting for specific materials, systems and methods

We require information from authors about some types of materials, experimental systems and methods used in many studies. Here, indicate whether each material, system or method listed is relevant to your study. If you are not sure if a list item applies to your research, read the appropriate section before selecting a response.

Materials & experimental systems

n/a	Involved in the study
☒	☐ Antibodies
☐	☒ Eukaryotic cell lines
☒	☐ Palaeontology and archaeology
☐	☒ Animals and other organisms
☒	☐ Clinical data
☒	☐ Dual use research of concern
☒	☐ Plants

Methods

n/a	Involved in the study
☒	☐ ChIP-seq
☒	☐ Flow cytometry
☒	☐ MRI-based neuroimaging

Eukaryotic cell lines

Policy information about cell lines and Sex and Gender in Research

Cell line source(s)	Yarrowia lipolytica W-29 derived terpenoid platform strain, provided by Prof Irina Borodina
Authentication	None of the cell lines were authenticated
Mycoplasma contamination	Cell lines were not tested for mycoplasma contamination
Commonly misidentified lines (See ICLAC register)	*Name any commonly misidentified cell lines used in the study and provide a rationale for their use.*

Animals and other research organisms

Policy information about studies involving animals; ARRIVE guidelines recommended for reporting animal research, and Sex and Gender in Research

Laboratory animals	Apis mellifera, of all ages.
Wild animals	The study did not involve wild animals.
Reporting on sex	Male and female honey bee pupae were used for sterol analysis. Only female bees were used in feeding trials.
Field-collected samples	No wild bees were collected in this study.
Ethics oversight	No ethical approval is required for working with honeybees in the UK. Small hives were used to minimise the number of bees that were required for these experiments. Whilst it was necessary to contain the hives used during feeding trials to prevent foraging on floral pollen, the hives were housed in a facility equipped with a cooling system, space for flight and excretion, as well as sugar and water supplies to minimise distress. The duration and frequency of hive inspections was kept to a minimum. Only samples relevant to the study were collected. Only sufficient samples for experimental robustness were collected. Samples were euthanised rapidly after collection to minimise distress.

Note that full information on the approval of the study protocol must also be provided in the manuscript.
